# Supplementary material for: shaPRS: Leveraging shared genetic effects across traits or ancestries improves accuracy of polygenic scores
Source: Am J Hum Genet. 2024 May 3;111(6):1006–17. doi: 10.1016/j.ajhg.2024.04.009 (PMC11179256; doi:10.1016/j.ajhg.2024.04.009)
Supplement: Document S1. Figures S1–S5, Tables S1, supplemental notes, and supplemental materials and methods [file mmc1.pdf]

The American Journal of Human Genetics, Volume *111*

Supplemental information

shaPRS: Leveraging shared genetic effects across traits or ancestries improves accuracy of polygenic scores

Martin Kelemen, Elena Vigorito, Laura Fachal, Carl A. Anderson, and Chris Wallace

Supplemental notes

shaPRS walkthrough examples

The following three examples may help to explain the application of shaPRS in three illustrative scenarios of SNPs: fully shared, non-shared and partially shared effect between studies.

Fully shared SNP. A SNP whose effect is 100% shared between proximal and adjunct studies. These are frequently null SNPs that have a true effect size of 0 ($\beta_1 = \beta_2 = \beta_{12} = 0$). Here, the estimated lFDR would be close to 1 (π=1), so the shaPRS equation

$$\beta_{shaPRS} = (1 - \pi)\beta_1 + \pi\beta_{12},$$

would simplify to

$$\beta_{shaPRS} \backsimeq 1 * \beta_{12}.$$

Thus here the final SNP estimate would become close to identical to the meta-analysis (β_{12}).

SNP effect is specific to proximal study. A SNP that only has an effect in the proximal study, but not in the adjunct study. An example of this would be some SNPs in the *NOD2* region in our IBD analyses, which are associated with CD susceptibility but not UC susceptibility. Here, the estimated lFDR would be 0 (π=0). Therefore, the shaPRS equation would simplify to

$$\beta_{shaPRS} \backsimeq 1 * \beta_1.$$

Thus, the final SNP estimate would become close to the proximal study (β_1).

SNP effect is partially shared between studies. A SNP that has an effect on both phenotypes, but with different magnitudes in the proximal and adjunct studies (which should give rise to a low lFDR for the variant's Cochran's test). Here, the estimated lFDR would be x (π=x), which would be a value between zero and one. Therefore, the shaPRS equation would become

$$\beta_{shaPRS} = (1 - x)\beta_1 + x\beta_{12}.$$

Thus, β_{shaPRS} has an intermediate value between the proximal effect and the meta-analysis effect that depends on the exact degree of effect sharing, specific to that particular SNP.

Missing SNPs and practical application of shaPRS

By default, the shaPRS R package will keep SNPs that are missing in the adjunct data by using their proximal data estimates, which is expected to produce the best overall quality PRS in practical applications. However, for our comparisons we excluded all SNPs that were missing from the adjunct datasets to ensure all methods worked from the same set of SNPs. Incorporating estimates from proximal-only SNPs would have had the effect of adding a constant value to PRS estimates from all methods, which would not have altered the rank order of the methods.

Supplemental figures and legends

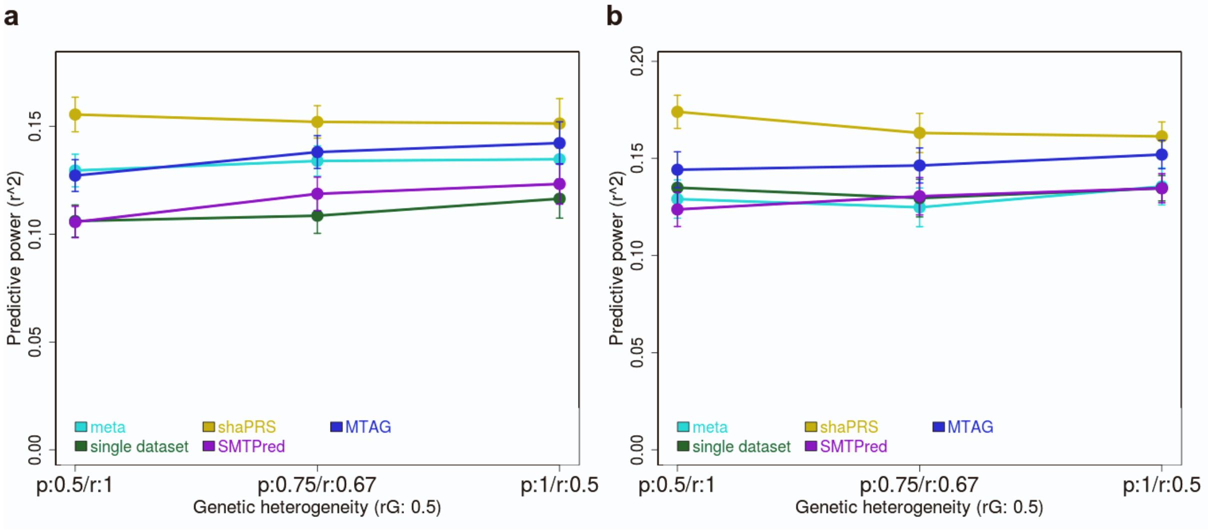

Fig S1: The effect of varying the composition of heterogeneity in the genetic correlation (the proportion of shared causal effects to their correlation) across the five methods. The X-axis shows the three different compositions that were used to generate the same genetic correlation (rG = 0.5). The axis labels are coded as *p/r*, which are the shared fraction of causal SNPs / effect size correlation of these SNPs. The Y-axis represents the squared correlation between the predicted and observed phenotypes on the test set and the error bars represent the standard error of the mean. Meta-analysis (blue) represents the PRS built from combining both phenotypes. 'Single dataset' (green) represents the PRS built from only the individuals from the proximal dataset. shaPRS (yellow) is our method, MTAG (blue) is a method that generates PRS by estimating SNP effect sizes based on constant parameters, and SMTPred (purple) is a method that produces a PRS by balancing the PRS for proximal and adjunct datasets based on their genetic correlation. **a.** Simulation scenario without the extra heterogeneity created by SNPs of large effect. **b.** The same simulation scenario as **a**, with the addition of the extra heterogeneity created by SNPs of large effect which contributed 5% non-shared heritability.

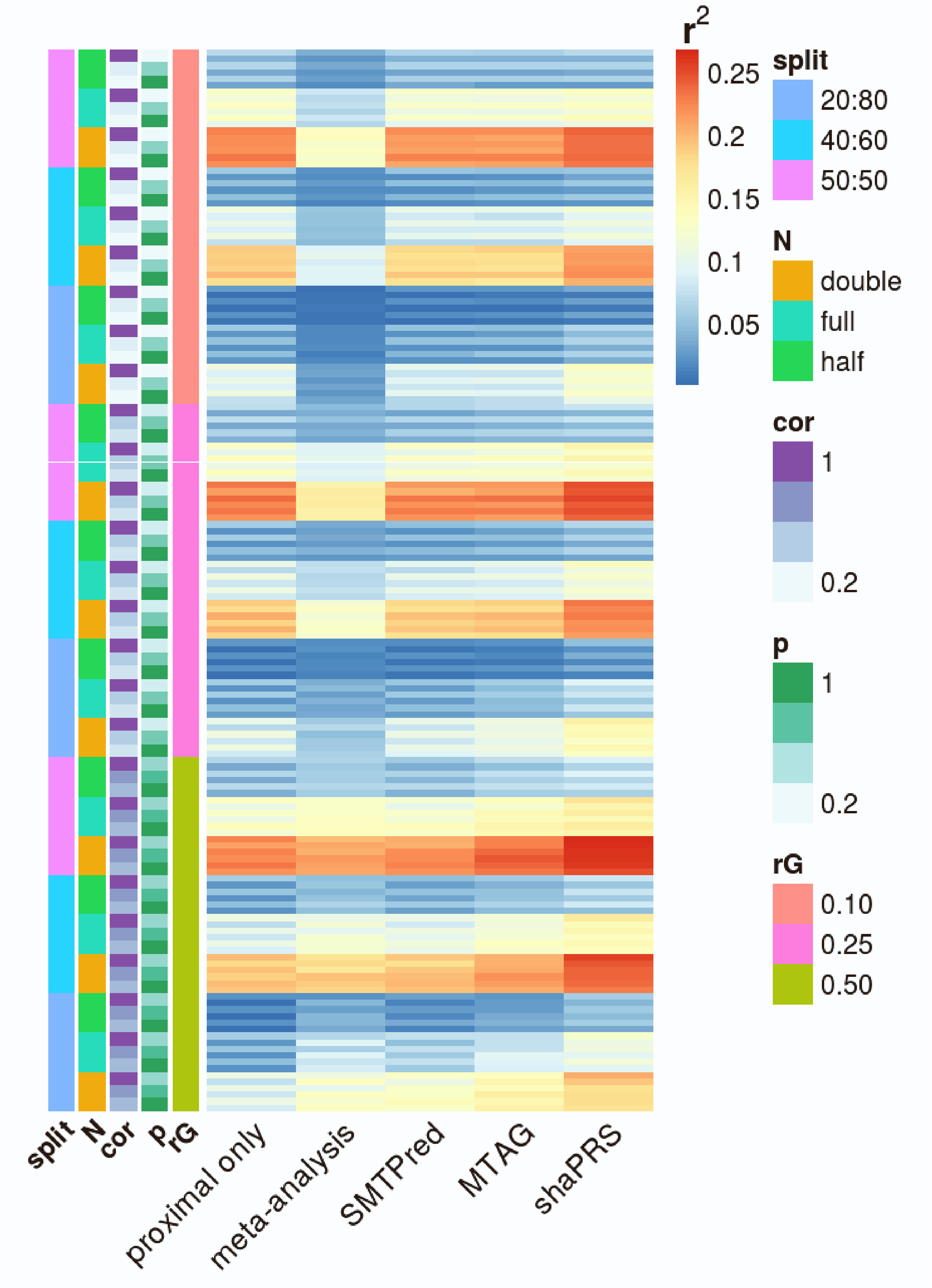

Fig S2: Heatmap of results for all 162 simulation scenarios, colours indicate the squared correlation between simulated and predicted phenotypes for example scenarios, where warmer colours indicate better performance. *N* is the sample size, half, full and double are 7,022, 14,044 and 28,088 individuals, respectively. *p* is the fraction of causal SNPs shared between the proximal and adjunct datasets, *cor* is the correlation of effect sizes between these SNPs. *split* is the ratio of the proximal to adjunct dataset sizes. *rG* is the genetic correlation between the proximal to adjunct datasets.

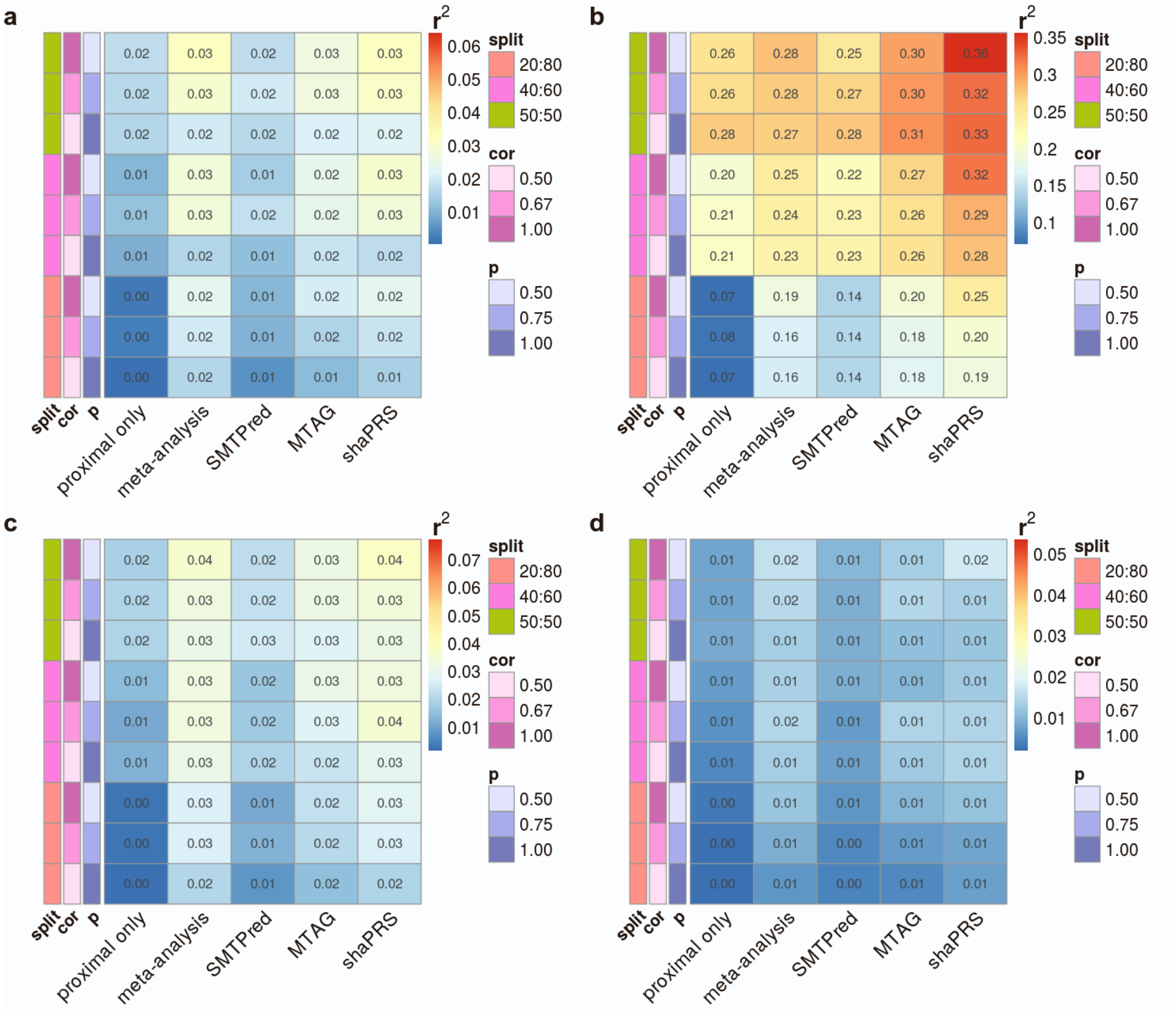

Fig S3: Heatmap of the squared correlation between simulated and predicted phenotypes in the additional 36 scenarios exploring selected parameters. *p* is the fraction of causal SNPs shared between the proximal and adjunct datasets, *cor* is the correlation of effect sizes between these SNPs. *split* is the ratio of the proximal to adjunct dataset sizes. Warmer colours indicate better performance. **a.** Sample size N = 14,044, with a proximal/adjunct sample ratio of 50/50, 40/60 or 20/80, a genetic correlation between proximal and adjunct traits of 0.5 with a heritability of 0.25 from 1,000 causal variants, no extra heterogeneity created by SNPs of large effect. **b.** The same scenario as **a**, with a heritability of 0.75. **b.** **c.** The same scenario as **b**, with a heritability of 0.5 and 3,000 causal SNPs. **d.** The same scenario as **c**, with 5,000 causal SNPs.

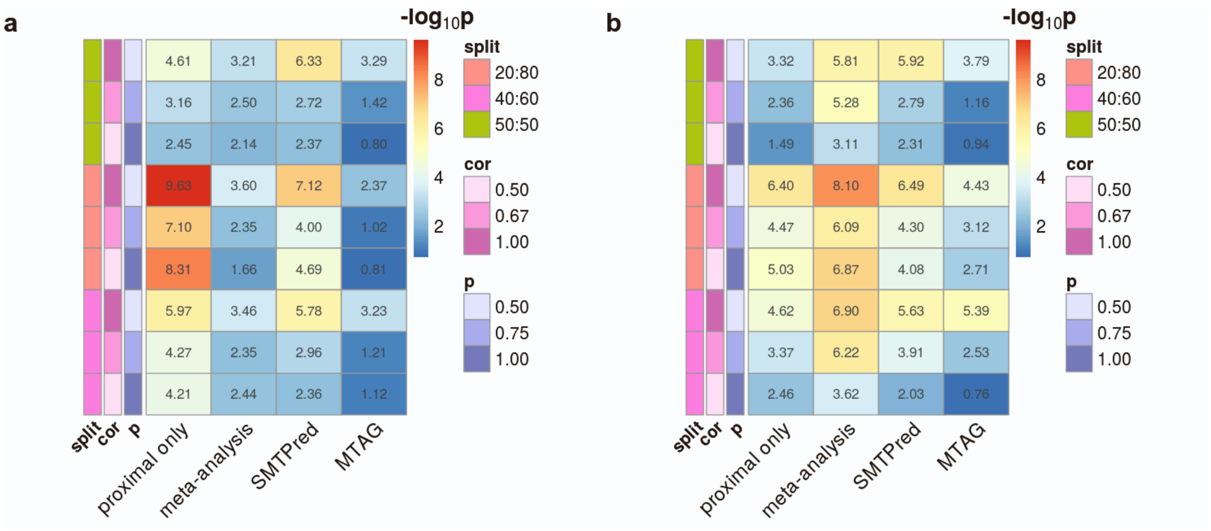

Fig S4: Heatmap of the median 'r2diff' -log10(p) model difference between shaPRS and other methods between simulated and predicted phenotypes for selected cross-trait genetic relationships. Warmer colours indicate stronger evidence for a difference between methods. **a.** A genome-wide genetic correlation between proximal and adjunct traits of 0.5 with a heritability of 0.5 from 1,000 causal variants and no extra heterogeneity created by SNPs of large effect. Sample size N = 14,044, with a proximal/adjunct sample ratio of 50/50, 40/60 or 20/80, and where *cor* is the correlation of effect sizes between SNPs and *P (or* $causal_S$*)* is the fraction of causal SNPs shared between the proximal and adjunct datasets. *split* is the ratio of the proximal to adjunct dataset sizes. **b.** The same scenario as **a**, with the addition of extra heterogeneity created by five SNPs of large effect that contributed 5% non-shared heritability. Results across the complete set of simulated scenarios are shown in Fig S2.

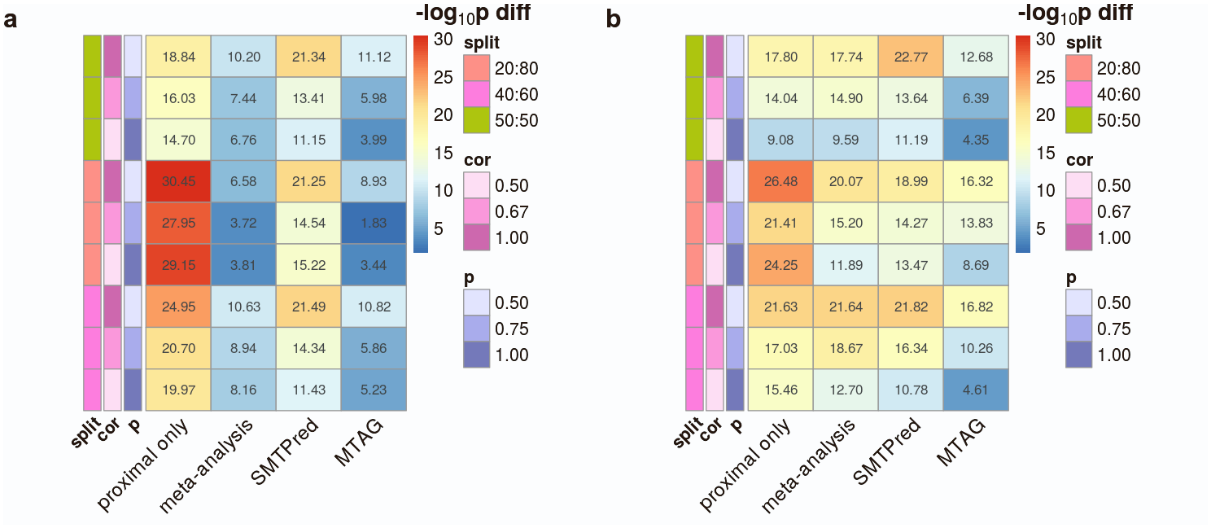

Fig S5: Heatmap of the difference between median -log10(p) values of the likelihood ratio tests of the complex model (which included both shaPRS and the other method) versus the nested model that included either just shaPRS or just the other method for selected cross-trait genetic relationships. Positive values and warmer colours indicate stronger evidence for improving performance by adding shaPRS into the model versus adding the other method. **a.** A genome-wide genetic correlation between proximal and adjunct traits of 0.5 with a heritability of 0.5 from 1,000 causal variants and no extra heterogeneity created by SNPs of large effect. Sample size N = 14,044, with a proximal/adjunct sample ratio of 50/50, 40/60 or 20/80, and where *cor* is the correlation of effect sizes between SNPs and *P (or* $causal_S$*)* is the fraction of causal SNPs shared between the proximal and adjunct datasets. *split* is the ratio of the proximal to adjunct dataset sizes. **b.** The same scenario as **a**, with the addition of extra heterogeneity created by five SNPs of large effect that contributed 5% non-shared heritability. Results across the complete set of simulated scenarios are shown in Fig S2.

Supplemental tables

Table S1 | Range of parameters evaluated in the simulation experiments.

parameter	range		
sample size	7,022, 14,044 and 28,088 training individuals		
phenotype split (proximal/adjunct)	50/50, 40/60 and 20/80		
five large effect SNPs	enabled or disabled		
	**rG**	**shared fraction of causal SNPs**	**effect size correlation**
rG composition	0.1	0.1	1
		0.55	0.182
		1	0.1
	0.25	0.25	1
		0.625	0.4
		1	0.25
	0.5	0.5	1
		0.75	0.667
		1	0.5

Sample size represents the number of individuals used for training the PRS, which were chosen to be approximately half, equal to or double the size of our UC GWAS datasets (N = 4,647 cases and 10,308 controls). Phenotype split represents the percentage of the samples with quantitative phenotypes simulated for each of the two traits, given as proximal/adjunct. The *'five large effect SNPs'* represents the choice to include five highly penetrant SNPs that explained 5% of the non-shared heritability of each trait. *rG* composition represents the different ways genetic correlations were constructed as a product of three different 'shared fraction of causal SNPs' and 'effect size correlation' estimates.

Table S2 | Leveraging information from GWAS studies with different ancestries

Target ancestry	trait	data	information pooling	PRS	r^2	AUC
EUR	T2D	proximal (EUR)	*N/A*	LDpred2	0.0075	0.624
				PRS-CS	0.00958	0.639
		proximal + adjunct	PRS-CSx	PRS-CSx	0.0125	0.658
				PRS-CSx-stage1	0.0114	0.650
			shaPRS	LDpred2	**0.0129**	**0.661**
				PRS-CS	0.0127	0.659
EUR	height	proximal	*N/A*	LDpred2	0.0976	*N/A*
				PRS-CS	0.116	
		proximal + adjunct	PRS-CSx	PRS-CSx	**0.123**	
				PRS-CSx-stage1	0.121	
			shaPRS	LDpred2	0.122	
				PRS-CS	0.122	
EUR	BRCA	proximal	*N/A*	LDpred2	0.00828	0.599
				PRS-CS	0.00529	0.584
		proximal + adjunct	PRS-CSx	PRS-CSx	0.00836	0.602
				PRS-CSx-stage1	0.00684	0.593
			shaPRS	LDpred2	**0.00955**	**0.607**
				PRS-CS	0.00724	0.596
EUR	CAD	proximal	*N/A*	LDpred2	0.00666	0.605
				PRS-CS	**0.0195**	**0.676**
		proximal + adjunct	PRS-CSx	PRS-CSx	0.0179	0.670
				PRS-CSx-stage1	0.0179	0.670
			shaPRS	LDpred2	0.0164	0.663
				PRS-CS	0.0169	0.665
		proximal	*N/A*	LDpred2	0.0115	0.595
				PRS-CS	0.0099	0.588
		proximal	PRS-CSx	PRS-CSx	0.0134	0.603
				PRS-CSx-stage1	0.0113	0.595

EUR	asthma	+ adjunct	shaPRS	LDpred2	**0.0136**	**0.604**
				PRS-CS	0.0123	0.599
AFR	BMI	proximal	*N/A*	LDpred2	0.0025	*N/A*
				PRS-CS	0.0037	
		proximal + adjunct	PRS-CSx	PRS-CSx	0.0210	
				PRS-CSx-stage1	0.0053	
			shaPRS	LDpred2	0.0196	
				PRS-CS	**0.0249**	
AFR	height	proximal	*N/A*	LDpred2	0.0035	*N/A*
				PRS-CS	0.0039	
		proximal + adjunct	PRS-CSx	PRS-CSx	**0.0282**	
				PRS-CSx-stage1	0.0091	
			shaPRS	LDpred2	0.0126	
				PRS-CS	0.0039	
AFR	LDL	proximal	*N/A*	LDpred2	0.0559	*N/A*
				PRS-CS	0.0082	
		proximal + adjunct	PRS-CSx	PRS-CSx	**0.0563**	
				PRS-CSx-stage1	0.0296	
			shaPRS	LDpred2	0.0208	
				PRS-CS	0.0187	

Table of the results of the cross-ancestry analysis that compared the accuracy of six different methods to produce a PRS. **Target ancestry** is the genetic ancestry of the target individuals on whom the final PRS was evaluated. **trait** is the phenotype evaluated. **data** is the summary statistic dataset used for training. Proximal is the GWAS conducted in the target ancestry and proximal+adjunct is the target and adjunct GWAS together. The adjunct GWAS was sourced from Japanese individuals in the case of European target PRS, and European individuals in the case of African target PRS. **information pooling** is the method that was used to pool the information from the proximal and adjunct datasets. *N/A* is when no information pooling took place, *PRS-CSx* is the PRS-CSx method and *shaPRS* is the method presented in this paper. **PRS** is the method that was used to generate the final PRS profiles. *LDpred2* is the PRS generated by the LDpred2-auto method that uses no additional genotype data. *PRS-CS* is the PRS generated by the PRS-CS method that uses no additional genotype data. *PRS-CSx* is the PRS generated by the PRS-CSx method that used validation data to weigh between the target and adjunct PRS. *PRS-CSx-stage1* is the PRS generated by the PRS-CSx method that did not use validation data to weigh between the target and adjunct PRS. **r^2** is the squared Pearson correlation coefficient between predicted and observed phenotypes. **AUC** is the area under the receiver operating characteristic curve of the predicted and observed phenotypes. All PRS were evaluated on strictly non-overlapping subsets of the UK Biobank.

Table S3 | shaPRS performance (r^2) relative to other methods in IBD subtypes

Other method	CD	UC
proximal	0.103 (-4%)	0.052 (-22%)
meta-analysis	0.095 (-12%)	0.061 (-6%)
SMTPred	0.100 (-7%)	0.059 (-10%)
MTAG	0.096 (-11%)	0.044 (-39%)
shaPRS	0.107 *(N/A)*	0.065 *(N/A)*

Table of the results for the inflammatory bowel disease subtype analysis that shows the performance improvements achieved by shaPRS relative to other methods. The values in each row are r^2, the squared Pearson correlation coefficient between predicted and observed phenotypes, followed by the percentage difference relative to shaPRS. **CD** is Crohn's disease and **UC** is ulcerative colitis.

Table S4A | CD - shaPRS comparison against other methods

	model difference		LRT of nested vs complex model (p-value)	
**method**	**r2redux p**	**Delong p**	**other**	**shaPRS**
**SMTPred**	0.041	0.23	0.055	2.290E-09
**MTAG**	0.053	0.041	4.550E-07	3.69E-18

Table S4B | UC- shaPRS comparison against other methods

	model difference		LRT of nested vs complex model (p-value)	
**method**	**r2redux p**	**Delong p**	**other**	**shaPRS**
**SMTPred**	0.029	0.044	0.171	1.530E-08
**MTAG**	2.440E-06	5.310E-06	0.138	1.200E-24

Results from the formal evaluation of model difference between shaPRS and other methods for the inflammatory bowel disease subtype. The **model difference** column shows the p-values if there was a difference between shaPRS and the other methods via the ‘r2redux r_diff’ and the pROC’ Delong’ tests, respectively. The **LRT of nested vs complex model** column shows the p-values for a likelihood ratio tests that evaluate if adding **shaPRS** or the **other** (non-shaPRS) PRS onto a nested model improves over the complex model of shaPRS+other. **A.** Crohn's disease and **B.** Ulcerative Colitis.

Table S5A | EUR-EAS asthma - shaPRS comparison against other methods				
		**method**	**PRS-CSx**	**PRS-CSx-stage1**
**model difference**	**shaPRS-PRSCS**	**r2redux p**	9.420E-07	0.0026
		**delong p**	3.290E-06	0.00257
	**shaPRS+LDpred2**	**r2redux p**	0.047	4.83E-13
		**delong p**	0.0947	5.64E-12
**LRT of nested vs complex model**	**shaPRS-PRSCS**	**other**	2.11E-37	7.420E-09
		**shaPRS**	0.00296	7.12E-32
	**shaPRS+LDpred2**	**other**	2.02E-16	3.260E-07
		**shaPRS**	9.32E-34	3.28E-81

Table S5B | EUR-EAS height - shaPRS comparison against other methods				
		**method**	**PRS-CSx**	**PRS-CSx-stage1**
**model difference**	**shaPRS-PRSCS**	**r2redux p**	1.100E-03	0.387
	**shaPRS+LDpred2**	**r2redux p**	0.821	0.127
**LRT of nested vs complex model**	**shaPRS-PRSCS**	**other**	2.66E-77	1.870E-56
		**shaPRS**	7.05E-34	6.50E-69
	**shaPRS+LDpred2**	**other**	<2.225074e-308	2.600E-297
		**shaPRS**	<2.225074e-308	<2.225074e-308

Table S5C | EUR-EAS T2D - shaPRS comparison against other methods

		method	PRS-CSx	PRS-CSx-stage1
model difference	shaPRS-PRSCS	r2redux p	0.533	6.567e-06
	shaPRS+LDpred2	r2redux p	0.219	2.359e-06
LRT of nested vs complex model	shaPRS-PRSCS	other	1.114e-17	1.106e-11
		shaPRS	1.588e-24	6.481e-56
	shaPRS+LDpred2	other	1.305e-28	1.729e-16
		shaPRS	2.942e-43	1.816e-68

Table S5D | EUR-EAS CAD- shaPRS comparison against other methods

		method	PRS-CSx	PRS-CSx-stage1
model difference	shaPRS-PRSCS	r2redux p	0.005	0.005
	shaPRS+LDpred2	r2redux p	2.992e-04	3.067e-4
LRT of nested vs complex model	shaPRS-PRSCS	other	7.982e-36	5.069e-36
		shaPRS	5.136e-13	3.451e-13
	shaPRS+LDpred2	other	7.314e-53	4.798e-53
		shaPRS	1.251e-17	8.696e-18

Table S5E | EUR-EAS BRCA- shaPRS comparison against other methods

		method	PRS-CSx	PRS-CSx-stage1
model difference	shaPRS-PRSCS	r2redux p	1.069e-05	0.217
	shaPRS+LDpred2	r2redux p	0.001	7.501e-10
LRT of nested vs complex model	shaPRS-PRSCS	other	2.279e-21	7.383e-07
		shaPRS	0.619	5.113e-15
	shaPRS+LDpred2	other	1.477e-05	0.002
		shaPRS	4.290e-28	1.003e-53

Table S5F | EUR-AFR BMI - shaPRS comparison against other methods

		method	PRS-CSx	PRS-CSx-stage1
model difference	shaPRS-PRSCS	r2redux p	1.590E-01	0.00164
	shaPRS+LDpred2	r2redux p	0.915	0.0207
LRT of nested vs complex model	shaPRS-PRSCS	other	7.11E-03	1.290E-03
		shaPRS	1.49E-07	2.62E-16
	shaPRS+LDpred2	other	2.25E-06	8.810E-04
		shaPRS	8.98E-07	2.33E-12

Table S5G | EUR-AFR height - shaPRS comparison against other methods

		method	PRS-CSx	PRS-CSx-stage1
model difference	shaPRS-PRSCS	r2redux p	9.810E-03	0.25
	shaPRS+LDpred2	r2redux p	0.000104	0.847
LRT of nested vs complex model	shaPRS-PRSCS	other	4.16E-09	2.32E-06
		shaPRS	2.69E-01	8.410E-11
	shaPRS+LDpred2	other	1.47E-12	3.810E-07
		shaPRS	9.96E-01	7.73E-08

Table S5H | EUR-AFR LDL - shaPRS comparison against other methods

		method	PRS-CSx	PRS-CSx-stage1
model difference	shaPRS-PRSCS	r2redux p	1.130E-11	0.00494
	shaPRS+LDpred2	r2redux p	9.81E-09	0.0475
LRT of nested vs complex model	shaPRS-PRSCS	other	1.83E-30	4.04E-15
		shaPRS	7.94E-01	1.170E-03
	shaPRS+LDpred2	other	1.01E-27	1.620E-13
		shaPRS	2.97E-01	4.11E-05

Table of the results for the formal evaluation of model difference between shaPRS and other methods for the cross-ancestry analyses. The **model difference** row shows the p values if there was a difference between shaPRS and the other methods via the ‘r2redux’ r_diff’ and for binary traits, the pROC’ Delong’ tests, respectively. The **LRT of nested vs complex model** row shows the p-values for a likelihood ratio tests that evaluate if adding **shaPRS** or the **other** PRS onto a nested model improves over the complex model of shaPRS+other. For these cross-ancestry analyses shaPRS was evaluated via both PRS-CS (**shaPRS-PRSCS**) and via LDpred2 (**shaPRS+LDpred2**). **A.** EUR-EAS asthma, **B.** EUR-EAS height, **C.** EUR-EAS T2D, **D.** EUR-EAS CAD, **E.** EUR-EAS BRCA, **F.** EUR-AFR BMI, **G.** EUR-AFR height and **H.** EUR-AFR LDL.

Supplementary Methods: mathematical derivations

1 Test for homogeneity of effects between two studies

Assume we have summary statistics for the same SNP from two studies, $\hat{\beta}_1, \hat{\beta}_2$ estimating the log odds ratios, and σ_1^2, σ_2^2 estimating their variances. We wish to test

$$H_0 : \beta_1 = \beta_2$$

where β_1, β_2 are the estimands of $\hat{\beta}_1, \hat{\beta}_2$.

Now

$$\mathrm{Var}(\hat{\beta}_1 - \hat{\beta}_2) = \sigma_1^2 + \sigma_2^2 - 2\rho\sigma_1\sigma_2$$

where

$$\rho = cor(\hat{\beta}_1, \hat{\beta}_2) = \frac{m_{12}\sqrt{n_1 n_2}}{\sqrt{m_1 m_2 (n_1 + m_1)(n_2 + m_2)}} \tag{1}$$

may be estimated using approximations derived by Lin and Sullivan (2009), with n_1, n_2 the number of cases in studies 1 and 2, m_1, m_2 the number of controls, and m_{12} the number of controls shared between both studies.

Thus a test statistic for homogeneity of effects at the same SNP in two different studies is

$$X^2 = \frac{(\hat{\beta}_1 - \hat{\beta}_2)^2}{\sigma_1^2 + \sigma_2^2 - 2\rho\sigma_1\sigma_2} \sim \chi_1^2$$

This is equivalent to Cochran's Q statistic for two studies, without assuming independence between the studies.

2 shaPRS summary effect estimate

shaPRS requires calculating the combined effect estimate if the two studies had been jointly analysed. We use the standard fixed effects meta analysis to give

$$\hat{\beta}_{12} = \frac{\hat{\beta}_1\sigma_1^{-2} + \hat{\beta}_2\sigma_2^{-2}}{\sigma_1^{-2} + \sigma_2^{-2}} = \frac{\hat{\beta}_1\tau_1 + \hat{\beta}_2\tau_2}{\tau_1 + \tau_2}$$

where to simplify the notation we use the conventional notation for the precision $\tau_. = 1/\sigma_.^2$ with

$$\begin{aligned}\sigma_{12}^2 = \mathrm{Var}(\hat{\beta}_{12}) &= \frac{\tau_1^2\sigma_1^2 + \tau_2^2\sigma_2^2 + \tau_1\tau_2\,\mathrm{cov}(\hat{\beta}_1, \hat{\beta}_2)}{(\tau_1 + \tau_2)^2} \\ &= \frac{\tau_1 + \tau_2 + \rho\sqrt{\tau_1\tau_2}}{(\tau_1 + \tau_2)^2}\end{aligned}$$

From this and the single study effects, a summary effect is estimated as

$$\bar{\beta} = w\hat{\beta}_1 + (1-w)\hat{\beta}_{12}$$

where $0 < w < 1$ is the lFDR. We have

$$\begin{aligned}
\text{cov}(\hat{\beta}_1, \hat{\beta}_{12}) &= \text{cov}\left(\hat{\beta}_1, \frac{\tau_1\hat{\beta}_1 + \tau_2\hat{\beta}_2}{\tau_1+\tau_2}\right) \\
&= \left(\frac{\tau_1}{\tau_1+\tau_2}\right)\text{cov}(\hat{\beta}_1, \hat{\beta}_1) + \left(\frac{\tau_2}{\tau_1+\tau_2}\right)\text{cov}(\hat{\beta}_1, \hat{\beta}_2) \\
&= \frac{1}{\tau_1+\tau_2} + \frac{\sqrt{\tau_2/\tau_1}}{\tau_1+\tau_2}\rho
\end{aligned}$$

so

$$\begin{aligned}
\bar{\sigma}^2 = \text{Var}\,\bar{\beta} &= w^2\,\text{Var}\,\hat{\beta}_1 + (1-w)^2\,\text{Var}\,\hat{\beta}_{12} + 2w(1-w)\,\text{cov}(\hat{\beta}_1, \hat{\beta}_{12}) \\
&= w^2/\tau_1 + (1-w)^2\frac{\tau_1+\tau_2+\rho\sqrt{\tau_1\tau_2}}{(\tau_1+\tau_2)^2} + 2w(1-w)\frac{1+\rho\sqrt{\tau_2/\tau_1}}{\tau_1+\tau_2} \\
&= \frac{w^2}{\tau_1} + \frac{1-w^2}{\tau_1+\tau_2} + \frac{(1-w)\sqrt{\tau_2}}{(\tau_1+\tau_2)^2\sqrt{\tau_1}}((\tau_1+2\tau_2)w+\tau_1)\rho
\end{aligned}$$

3 Correlation between shaPRS summary effect at two SNPs

In order to calculate a PRS we also need an estimate of the correlation between shaPRS summary effects $\bar{\beta}_A, \bar{\beta}_B$ at two SNPs in LD, A and B, for LD-aware polygenic score methods. From here on we will use subscripts A, B to denote quantities relating to SNPs A and B, and $1, 2$ to denote quantities relating to studies 1 and 2, possibly in combination.

$$\begin{aligned}
\text{cov}(\bar{\beta}_A, \bar{\beta}_B) &= cov\left[\left(w_A\hat{\beta}_{A1} + (1-w_A)\hat{\beta}_{A12}\right), \left(w_B\hat{\beta}_{B2} + (1-w_B)\hat{\beta}_{B12}\right)\right] \\
&= cov\left[\left(w_A\hat{\beta}_{A1} + (1-w_A)\frac{\tau_{A1}\hat{\beta}_{A1}+\tau_{A2}\hat{\beta}_{A2}}{\tau_{A1}+\tau_{A2}}\right), \left(w_B\hat{\beta}_{B2} + (1-w_B)\frac{\tau_{B1}\hat{\beta}_{B1}+\tau_{B2}\hat{\beta}_{B2}}{\tau_{B1}+\tau_{B2}}\right)\right] \\
&= \left(\frac{\tau_{A1}+w_A\tau_{A2}}{\tau_{A1}+\tau_{A2}}\right)\left(\frac{\tau_{B1}+w_B\tau_{B2}}{\tau_{B1}+\tau_{B2}}\right)\text{cov}(\hat{\beta}_{A1}, \hat{\beta}_{B1}) + \\
&\quad \left(\frac{\tau_{A1}+w_A\tau_{A2}}{\tau_{A1}+\tau_{A2}}\right)\left(\frac{(1-w_B)\tau_{B2}}{\tau_{B1}+\tau_{B2}}\right)\text{cov}(\hat{\beta}_{A1}, \hat{\beta}_{B2}) + \\
&\quad \left(\frac{(1-w_A)\tau_{A2}}{\tau_{A1}+\tau_{A2}}\right)\left(\frac{\tau_{B1}+w_B\tau_{B2}}{\tau_{B1}+\tau_{B2}}\right)\text{cov}(\hat{\beta}_{A2}, \hat{\beta}_{B1}) + \\
&\quad \left(\frac{(1-w_A)\tau_{A2}}{\tau_{A1}+\tau_{A2}}\right)\left(\frac{(1-w_B)\tau_{B2}}{\tau_{B1}+\tau_{B2}}\right)\text{cov}(\hat{\beta}_{A2}, \hat{\beta}_{B2}) \qquad (2)
\end{aligned}$$

Standard results give covariances for different SNPs in the same study (Burren *et al.*, 2014)

$$\text{cov}(\hat{\beta}_{A1}, \hat{\beta}_{B1}) = r_1/\sqrt{\tau_{A1}\tau_{B1}}$$
$$\text{cov}(\hat{\beta}_{A2}, \hat{\beta}_{B2}) = r_2/\sqrt{\tau_{A2}\tau_{B2}}$$

where r_i denotes the correlation between genotypes at A and B in study i, or the same SNPs in different studies (Lin and Sullivan, 2009)

$$\text{cov}(\hat{\beta}_{A1}, \hat{\beta}_{A2}) = \rho/\sqrt{\tau_{A1}\tau_{B1}}$$
$$\text{cov}(\hat{\beta}_{B1}, \hat{\beta}_{B2}) = \rho/\sqrt{\tau_{A2}\tau_{B2}}$$

3.1 Non-overlapping samples between studies

If the different studies have no overlap, $\rho = 0$ and the covariance between estimates from different studies (same or different SNPs) is 0. From (2) then, in the case of no overlapping samples, but possibly different populations (so $r_1 \neq r_2$)

$$\text{cov}(\bar{\beta}_{A1}, \bar{\beta}_{B2}) = \left(\frac{\tau_{A1} + w_A\tau_{A2}}{\tau_{A1} + \tau_{A2}}\right)\left(\frac{\tau_{B1} + w_B\tau_{B2}}{\tau_{B1} + \tau_{B2}}\right)\frac{r_1}{\sqrt{\tau_{A1}\tau_{B1}}} + \left(\frac{(1 - w_A)\tau_{A2}}{\tau_{A1} + \tau_{A2}}\right)\left(\frac{(1 - w_B)\tau_{B2}}{\tau_{B1} + \tau_{B2}}\right)\frac{r_2}{\sqrt{\tau_{A2}\tau_{B2}}}$$

and

$$\begin{aligned}\text{cor}(\bar{\beta}_{A1}\bar{\beta}_{B2}) &= \frac{\text{cov}(\bar{\beta}_{A1}, \bar{\beta}_{B2})}{\sqrt{\bar{\sigma}_A\bar{\sigma}_B}} \\ &= \frac{\sqrt{\tau_{A1}\tau_{A2}\tau_{B1}\tau_{B2}}(1 - w_A)(1 - w_B)r_2 + (\tau_{A1} + w_A\tau_{A2})(\tau_{B1} + w_B\tau_{B2})r_1}{\sqrt{(\tau_{A1} + \tau_{A2})(\tau_{B1} + \tau_{B2})(\tau_{A1} + w_A^2\tau_{A2})(\tau_{B1} + w_B^2\tau_{B2})}}\end{aligned}$$

When the two studies reflect the same populations, so $r_1 = r_2 = r$,

$$\text{cor}(\bar{\beta}_{A1}\bar{\beta}_{B2}) = r \times \frac{\sqrt{\tau_{A1}\tau_{A2}\tau_{B1}\tau_{B2}}(1 - w_A)(1 - w_B) + (\tau_{A1} + w_A\tau_{A2})(\tau_{B1} + w_B\tau_{B2})}{\sqrt{(\tau_{A1} + \tau_{A2})(\tau_{B1} + \tau_{B2})(\tau_{A1} + w_A^2\tau_{A2})(\tau_{B1} + w_B^2\tau_{B2})}}$$

Note that this may be < 1.

3.2 Overlapping samples between studies

Where studies do have overlapping samples, we assume they are from the same population so $r_1 = r_2 = r$, and we follow the Appendix in Lin and Sullivan (2009) to estimate covariances between different SNPs in different studies. Switching for now to the notation there `https://www.ncbi.nlm.nih.gov/pmc/articles/PMC2790578/#app1`, much of the same results hold, and we have

$$\text{cov}(\hat{\theta}_k, \theta_l) \simeq I_k^{-1} \text{cov}\{U_k(\theta_k)U_l(\theta_l)\}I_l^{-1}(\theta_l) \tag{3}$$

where $\hat{\theta}_k$ is the root of the score function

$$U_k(\theta_k) = \sum_{i=1}^{N_k} \left(Y_i - \frac{e^{\alpha_k + \beta_k' X_i}}{1 + e^{\alpha_k + \beta_k' X_i}} \right) \tilde{X}_i$$

where N_k is the total sample size, $\tilde{X}_i$ is the covariate matrix augmented with a column of 1s and $\mathrm{Var}(\theta_k) \simeq I_k^{-1}(\theta_k)$ with

$$I_k(\theta_k) = \sum_{i=1}^{N_k} \frac{e^{\alpha_k + \beta_k' X_i}}{(1 + e^{\alpha_k + \beta_k' X_i})^2} \tilde{X}_i \tilde{X}_i{}'$$

Unlike Lin and Sullivan (2009), j, k now refer to **different** SNPs in different studies (eg $j = A1, k = B2$), so $I_k \neq I_l$. Under the null hypothesis, we assume $\beta_k = \beta_l = 0$, allowing some simplification.

$$\begin{aligned}
I_k(\theta_k) &\simeq \frac{N_k e^{\alpha_k}}{(1 + e^{\alpha_k})^2} N_k^{-1} \begin{pmatrix} n_k & \sum X_{ki} \\ \sum X_{ki} & \sum X_{ki}^2 \end{pmatrix} \\
&\simeq \frac{N_k e^{\alpha_k}}{(1 + e^{\alpha_k})^2} \begin{pmatrix} 1 & 2f_k \\ 2f_k & 2f_k(1 + f_k) \end{pmatrix} \\
I_k^{-1}(\theta_k) &\simeq \frac{(1 + e^{\alpha_k})^2}{N_k e^{\alpha_k}} \left(\frac{1}{2f_k(1 + f_k)} - 4f_k^2 \right) \begin{pmatrix} 2f_k(1 + f_k) & -2f_k \\ -2f_k & 1 \end{pmatrix}
\end{aligned}$$

where n_k is cases in study k, f_k is the minor allele frequency for the SNP tested in study k, and similarly for I_l. The approximation to $\mathrm{cov}\{U_k(\theta_k), U_l(\theta_l)\}$ also differs

$$\begin{aligned}
\mathrm{cov}\{U_k(\theta_k), U_l(\theta_l)\} &\simeq \sum_i^{N_{lk}} \left(Y_i - \frac{e^{\alpha_k}}{1 + e^{\alpha_k}} \right) \left(Y_i - \frac{e^{\alpha_l}}{1 + e^{\alpha_l}} \right) \\
&\simeq \frac{e^{\alpha_k + \alpha_l}}{(1 + e^{\alpha_k})(1 + e^{\alpha_l})} \begin{pmatrix} N_{lk} & \sum X_{li} \\ \sum X_{ki} & \sum X_{li} X_{ki} \end{pmatrix} \\
&\simeq \frac{N_{lk} e^{\alpha_k + \alpha_l}}{(1 + e^{\alpha_k})(1 + e^{\alpha_l})} \begin{pmatrix} 1 & 2f_l \\ 2f_k & 2r\sqrt{f_k f_l (1 - f_k)(1 - f_l)} + 4f_k f_l \end{pmatrix}
\end{aligned}$$

where N_{lk} is the number of shared subjects between studies l, k. Thus from (3)

$$\mathrm{cov}(\hat{\theta}_k, \hat{\theta}_l) \simeq \frac{(1 + e^{\alpha_k})(1 + e^{\alpha_l}) N_{kl}}{4N_k N_l f_k f_l (1 - f_k)(1 - f_l)} \begin{pmatrix} 4f_k f_l (F + f_k f_l - f_k - f_l + 1) & -2F f_k \\ -2F f_l & F \end{pmatrix}$$

where $F = 2r\sqrt{f_k f_l (1 - f_k)(1 - f_l)}$ so

$$\begin{aligned}
\mathrm{cov}(\hat{\beta}_k, \hat{\beta}_l) &\simeq \frac{(1 + e^{\alpha_k})(1 + e^{\alpha_l}) N_{kl}}{4N_k N_l f_k f_l (1 - f_k)(1 - f_l)} 2r\sqrt{f_k f_l (1 - f_k)(1 - f_l)} \\
&= \frac{(1 + e^{\alpha_k})(1 + e^{\alpha_l}) N_{kl}}{2N_k N_l \sqrt{f_k f_l (1 - f_k)(1 - f_l)}} r
\end{aligned}$$

The MAF enter here because variance of the genotype is a function of MAF. We know (Giambartolomei *et al.*, 2014) that

$$\operatorname{Var}\hat{\beta}_k \simeq 1/\tau_k = \frac{(n_k + m_k)}{n_k m_k \times 2 f_k (1 - f_k)}$$

so we can substitute

$$f_k(1 - f_k) = \frac{(n_k + m_k)\tau_k}{2 n_k m_k}$$

and we also have $e^{\alpha_k} \simeq n_k/m_k$ (Lin and Sullivan, 2009) where m_k is the number of controls in study k, so that (returning to the notation used in this manuscript)

$$\begin{aligned}
\operatorname{cov}(\hat{\beta}_{A1}, \hat{\beta}_{B2}) &\simeq \frac{r m_{12}\sqrt{n_1 n_2}}{\sqrt{m_1 m_2 (n_1 + m_1)(n_2 + m_2)\tau_{A1}\tau_{B2}}} \\
\operatorname{cor}(\hat{\beta}_{A1}, \hat{\beta}_{B2}) &\simeq \frac{\operatorname{cov}(\hat{\beta}_{A1}, \hat{\beta}_{B2})}{\sqrt{\sigma^2_{A1}\sigma^2_{B1}}} \\
&\simeq \frac{r m_{12}\sqrt{n_1 n_2}}{\sqrt{m_1 m_2 (n_1 + m_1)(n_2 + m_2)}} \\
&\simeq r\rho
\end{aligned}$$

and similarly

$$\operatorname{cov}(\hat{\beta}_{A2}, \hat{\beta}_{B1}) \simeq \frac{r\rho}{\sqrt{\tau_{A2}\tau_{B1}}}$$

With this, from (2) we can easily estimate

$$\begin{aligned}
\operatorname{cov}(\bar{\beta}_A, \bar{\beta}_B) = &\left(\frac{\tau_{A1} + w_A\tau_{A2}}{\tau_{A1} + \tau_{A2}}\right)\left(\frac{\tau_{B1} + w_B\tau_{B2}}{\tau_{B1} + \tau_{B2}}\right)\frac{r}{\sqrt{\tau_{A1}\tau_{B1}}} + \\
&\left(\frac{\tau_{A1} + w_A\tau_{A2}}{\tau_{A1} + \tau_{A2}}\right)\left(\frac{(1 - w_B)\tau_{B2}}{\tau_{B1} + \tau_{B2}}\right)\frac{r\rho}{\sqrt{\tau_{A1}\tau_{B2}}} + \\
&\left(\frac{(1 - w_A)\tau_{A2}}{\tau_{A1} + \tau_{A2}}\right)\left(\frac{\tau_{B1} + w_B\tau_{B2}}{\tau_{B1} + \tau_{B2}}\right)\frac{r\rho}{\sqrt{\tau_{A2}\tau_{B1}}} + \\
&\left(\frac{(1 - w_A)\tau_{A2}}{\tau_{A1} + \tau_{A2}}\right)\left(\frac{(1 - w_B)\tau_{B2}}{\tau_{B1} + \tau_{B2}}\right)\frac{r}{\sqrt{\tau_{A2}\tau_{B2}}}
\end{aligned}$$

References

Burren, O. S., Guo, H., and Wallace, C. (2014). VSEAMS: A pipeline for variant set enrichment analysis using summary GWAS data identifies IKZF3, BATF and ESRRA as key transcription factors in type 1 diabetes. *Bioinformatics*, **30**(23), 3342–3348.

Giambartolomei, C., Vukcevic, D., Schadt, E. E., Franke, L., Hingorani, A. D., Wallace, C., and Plagnol, V. (2014). Bayesian Test for Colocalisation between Pairs of Genetic Association Studies Using Summary Statistics. *PLOS Genetics*, **10**(5), e1004383.

Lin, D.-Y. and Sullivan, P. F. (2009). Meta-analysis of genome-wide association studies with overlapping subjects. *Am. J. Hum. Genet.*, **85**(6), 862–872.
