# Supplementary material for: shaPRS: Leveraging shared genetic effects across traits or ancestries improves accuracy of polygenic scores
Source: Am J Hum Genet. 2024 May 3;111(6):1006–17. doi: 10.1016/j.ajhg.2024.04.009 (PMC11179256; doi:10.1016/j.ajhg.2024.04.009)
Supplement: Document S2. Article plus supplemental information [file mmc2.pdf]

ARTICLE

shaPRS: Leveraging shared genetic effects across traits or ancestries improves accuracy of polygenic scores

Authors

Martin Kelemen, Elena Vigorito, Laura Fachal, Carl A. Anderson, Chris Wallace

Correspondence

mk907@cam.ac.uk

We introduce shaPRS, a polygenic risk score (PRS) pre-processing method that improves predictive performance of PRSs by leveraging the genetic overlap between traits or ancestries. Importantly, shaPRS requires only GWAS summary statistics of two partially correlated traits or ancestries and is agnostic with respect to the method used to generate the PRSs.

Kelemen et al., 2024, The American Journal of Human Genetics *111*, 1006–1017

June 6, 2024 © 2024 The Authors.

https://doi.org/10.1016/j.ajhg.2024.04.009

CellPress

ARTICLE

shaPRS: Leveraging shared genetic effects across traits or ancestries improves accuracy of polygenic scores

Martin Kelemen,[1,2,*] Elena Vigorito,[3] Laura Fachal,[1] Carl A. Anderson,[1,4] and Chris Wallace[2,3,4]

Summary

We present shaPRS, a method that leverages widespread pleiotropy between traits or shared genetic effects across ancestries, to improve the accuracy of polygenic scores. The method uses genome-wide summary statistics from two diseases or ancestries to improve the genetic effect estimate and standard error at SNPs where there is homogeneity of effect between the two datasets. When there is significant evidence of heterogeneity, the genetic effect from the disease or population closest to the target population is maintained. We show via simulation and a series of real-world examples that shaPRS substantially enhances the accuracy of polygenic risk scores (PRSs) for complex diseases and greatly improves PRS performance across ancestries. shaPRS is a PRS pre-processing method that is agnostic to the actual PRS generation method, and as a result, it can be integrated into existing PRS generation pipelines and continue to be applied as more performant PRS methods are developed over time.

Introduction

Genome-wide association studies (GWASs) provide a routine means of quantifying the effects of genetic variation on human diseases and traits. One possible use of these genetic effect estimates is the creation of polygenic risk scores (PRSs), an approximation of an individual's genome-wide genetic propensity for a given trait or disease. Recent studies have shown that individuals in the upper extreme tail of polygenic risk for some common diseases have equivalent risk to those carrying monogenic mutations for these phenotypes.[1,2] Driven by these observations, there is hope that polygenic scores can be used alongside traditional clinical and demographic predictors of disease to diagnose disease earlier and with greater accuracy.[3,4]

Unfortunately, the clinical utility of polygenic scores is currently limited by the GWAS on which they are based. The precision with which GWASs can estimate genetic effects on disease risk increases with sample size. Recent studies have suggested that most complex diseases will require somewhere between a few hundred thousand to several million cases to accurately capture genome-wide genetic effects on disease risk.[5,6] As a result, the information content of all current GWASs is imperfect, reducing the accuracy of the polygenic scores generated from them. There is an expectation that GWAS meta-analyses across vast population biobanks will get us closer to quantifying SNP effects that fully capture heritability for some common complex diseases. However, many debilitating and life-threatening complex diseases have lower population prevalence, preventing even meta-analyses across large biobanks from ascertaining sufficient cases to facilitate the construction of accurate polygenic scores.

It is not only less common complex diseases that are set to be precluded from any clinical advantages brought about by polygenic scores. Genomics is failing on diversity.[7] On February 14th, 2024 the GWAS Diversity Monitor[8] showed that 94.51% of individuals included in GWASs were from European ancestries. Recent studies have demonstrated the poor portability of polygenic risk scores across populations due to differences in effect sizes and LD structure.[9] Migration events and population bottlenecks can lead to large differences in allele frequencies between ancestries, and as a result of the biased application of GWASs, we are missing accurate disease risk estimates for the many variants that are only common outside of European ancestry groups.[10,11] Thankfully, the clarion call for major improvements in the ancestral diversity of GWASs, and genomics studies more generally, is now loud.[7,12,13] Recent studies in non-Europeans have highlighted the advantages of increased diversity of GWASs, delivering both novel genetic associations and biological insights that were missed even in the larger European ancestry GWASs.[9,14–16] If PRSs do start to deliver on their hype, then further diversification cannot come soon enough—otherwise, we run the risk of widening existing health inequalities.

While genetic effects on disease certainly do differ between populations, many risk variants are believed to be shared across divergent ancestry groups.[17,18] There is also a growing appreciation of the extent to which genetic effects are shared across different disorders. For clinically and biologically related diseases such as Crohn disease (CD) and ulcerative colitis (UC), the two common forms

[1]Wellcome Sanger Institute, Hinxton, Cambridgeshire, UK; [2]Cambridge Institute of Therapeutic Immunology & Infectious Disease, University of Cambridge, Cambridge, UK; [3]MRC Biostatistics Unit, University of Cambridge, Cambridge, UK
[4]These authors contributed equally
*Correspondence: mk907@cam.ac.uk
https://doi.org/10.1016/j.ajhg.2024.04.009.
© 2024 The Authors. This is an open access article under the CC BY license (http://creativecommons.org/licenses/by/4.0/).

of inflammatory bowel disease, genetic effects are often shared. Across immune-mediated disease more generally, the number of known pleiotropic effects continues to grow, a phenomenon that is mirrored in other disease groups such as metabolic and psychiatric disorders. A principled pooling of information across traits[19,20] and ancestries[21–23] has already been shown to improve the prediction accuracy of PRSs. A common assumption of these methods Is that weights given to each dataset are constant across SNPs. In reality, this assumption is frequently violated, as the extent of sharing, either between two diseases or two populations, varies across SNPs.[24,25]

We introduce a method, shaPRS (pronounced "shapers"), a PRS pre-processing step that can be integrated into existing PRS generation pipelines that allows the integration of imperfectly shared information between two GWAS datasets. We assume one dataset is representative of the target population, hereafter referred to as the proximal dataset, and that a second adjunct dataset may provide relevant information, but that the degree of relevance varies across the genome. Our approach, which only requires summary statistics for each dataset, estimates weights that summarize how relevant the adjunct dataset is at each SNP to perform a weighted meta-analysis of the two datasets. We also generate a new pairwise SNP correlation matrix that captures the effect of this weighting and allows for partial sharing of controls and/or the use of distinct SNP correlation matrices for the two input studies (e.g., in the case of different ancestries). This matrix may be used together with the weighted SNP effect estimates in any downstream PRS software. We show via large-scale simulations in the UK Biobank (UKBB)[26] that shaPRS outperforms similar methods. We then apply shaPRS to nine real GWAS datasets to illustrate the improvements it brings to PRS accuracy, both across diseases and across ancestral populations.

Material and methods

Overview of method

shaPRS, which uses GWAS summary statistics, is a PRS pre-processing step based on a weighted meta-analysis of two partially related GWAS studies. We begin by testing, at each SNP, the evidence against homogeneity of effect between the two studies using Cochran's test. From these test statistics, we calculate the local false discovery rate (lFDR)[27] as an estimate of the probability that the estimates reflect the same "common truth." Where the lFDR is high, the datasets can likely be combined, and we favor β_{12}, which is the standard inverse variance weighted average of the effect estimates in the proximal study, β_1, and the adjunct study, β_2. We aim to minimize the variance of estimated effect sizes by including information from the adjunct study, where doing so is unlikely to cause bias. Where the lFDR is low, we are conservative, and favor β_1 from the proximal study, aiming to minimize bias at the expense of higher variance. We thus calculate a final shaPRS SNP effect estimate as

$$\beta_{shaPRS} = (1 - \pi)\beta_1 + \pi\,\beta_{12},$$

where π denotes the lFDR. As the use case of our method is a seamless integration into existing PRS generation pipelines, a full set of summary statistics are derived, including standard errors, p values, and sample size, as described in the material and methods section. An illustrative example is provided in the supplemental information.

The current generation of most performant PRS generation methods[28–30] also require an appropriate LD-matrix, often obtained from a reference panel. Therefore, to obtain an LD-reference panel appropriate for the derived summary statistics that represent information from the weighting and possibly LD from different ancestries, we provide a method to derive a new matrix describing the correlation between β_{shaPRS} across different SNPs (supplemental notes).

ShaPRS genetic association summary statistics blending

Our method favors the proximal dataset effect estimate β_1 where the effect estimates appear to differ between proximal and adjunct datasets, and the combined effect estimate β_{12} (the standard fixed effects meta-analysis estimate obtained from β_1 and the adjunct study coefficient β_2) when the effect estimates for the two datasets are similar. In other words, we choose the more precise proximal phenotype with lower bias where SNP effects are heterogeneous but prefer the larger sample size with lower variance where the SNP effects are congruent between single datasets.

To make this decision, we use Cochran's Q-test to assess heterogeneity of effects between the two datasets at each variant, modified to allow for shared controls between the cohorts.

$$Q = \frac{(\beta_1 - \beta_2)^2}{\sigma_1^2 + \sigma_2^2 - 2\rho\sigma_1\sigma_2}, Q \sim \chi^2 \qquad \text{(Equation 1)}$$

where $\boldsymbol{\sigma_1}/\boldsymbol{\sigma_2}$ are the standard errors for the proximal and adjunct datasets, respectively, and ρ is an estimate of the correlation between β_1 and β_2 obtained as a simple function of sample sizes.[31]

To estimate the probability that effects are heterogeneous, we used an lFDR approach, estimating

$$\pi = Pr(H_0|p),$$

where H_0 is the null hypothesis for the SNP, and p is the (adjusted) Q-test p value obtained from the Chi-squared distribution with 1° of freedom as defined above. The lFDR values were then estimated from these p values by the *qvalue* R package (https://github.com/StoreyLab/qvalue).

The blended effect estimate is then

$$\beta_{shaPRS} = \pi\beta_{12} + (1 - \pi)\beta_1.$$

The goal of our method is to generate a new, complete set of summary statistics that may be used by any downstream PRS generation tool. These statistics include a new set of SNP coefficients, their standard errors, and the correlation between coefficients. The supplemental notes set out derivations for the standard errors and correlation matrix, and functions to calculate these are provided in the R package (https://github.com/mkelcb/shaprs).

Simulation analyses

Our simulations relied on the UKBB cohort, which has been previously described in detail elsewhere.[26] We excluded individuals who were sex-discordant, not White British, or had third-degree relatives or closer in the cohort, as defined in the UKBB documentation. Genotype data were filtered to an intersection of the HapMap3 panel (a common practice for PRS generation, but not a requirement for shaPRS) and a subset that excluded variants with an INFO score <0.8, MAF <0.1%, missing genotype rate >2%, or deviated from Hardy-Weinberg equilibrium ($p < 10^{-7}$).

From this subset, we randomly chose 3,158 individuals to serve as a test set (approximately 20% of the size of our inflammatory bowel disease [IBD] dataset).

We evaluated the effect of cohort size by considering three studies with approximately half ($N = 7{,}022$), equal to ($N = 14{,}044$), or double ($N = 28{,}088$) the number of samples in our genotyped IBD cohort after withholding 3,158 individuals as a test set that were not used for model training. We also considered three different ratios to split our source samples into the two phenotypes (proximal and adjunct). These ratios were 20/80, 40/60, and 50/50 for phenotypes 1 and 2, respectively. Additionally, we varied the range of pleiotropic architectures considered by evaluating three genetic correlations (0.1, 0.25, and 0.5) made up of three combinations of shared and non-shared SNP effects. The motivation for the latter was to examine the key ability of our method to adapt to different compositions of shared and non-shared genetic effects that comprise a given level of genetic correlation. We considered three different scenarios (low, medium, and high, as defined in Table S1) of shared effects per genetic correlation, making up a total of nine arrangements. We also considered an additional scenario, where five SNPs contribute 5% of the total non-shared heritability for each trait. We evaluated all possible parameter combinations at a heritability of 0.5 arising from 1,000 causal variants for a set of 162 genetic architecture scenarios. We also evaluated the performance of all methods in the four additional scenarios where we have held all parameters at their base value except for one (the number of causal SNPs of 3,000 or 5,000 and a heritability of 0.25 or 0.75). The results of these additional 36 simulation scenarios can be found in Figure S3. We used LDAK 5.0[32] to simulate 20 replicates for bivariate quantitative phenotypes whose SNP effect sizes we generated via our custom R scripts according to the schema described above. We evaluated shaPRS's performance by comparing its predictive accuracy on the test set against four baselines: the single proximal dataset on its own, the meta-analysis of the proximal and adjunct datasets, and the SMTPred and MTAG methods. Both SMTPred and MTAG were trained directly on the PLINK summary statistics using their own Python functions *ldsc_wrapper.py* and *mtag.py* for SMTPred and MTAG, respectively. To accommodate the scale of our simulations, the final PRSs were generated via RapidoPGS, a computationally efficient PRS generation method.[33] To evaluate if using RapidoPGS had introduced any bias into our analyses, we re-generated the PRSs of 50 randomly selected replicates (10 for each method) with LDpred2-auto. For this, we chose the scenario involving 14,044 individuals, phenotypes divided 50/50, with a genetic correlation (rG) of 0.5 made up from half of the causal variants shared with a correlation of 1.0, without any highly penetrant variants. We found the relative performance of the methods did not change and that the results were strongly congruent between LDpred2 and RapidoPGS (Spearman rank correlation of 0.781).

Generating genome-wide summary statistics for CD and UC

The availability of all IBD datasets is described in the web resources section. The sample collection protocols are described in the original publications of each study.[34–36] Initial quality control procedures for the studies where the IBD PRS performance was tested are described in the original publications.[26,35,36] For the IBD training dataset and prior to genotype imputation, we excluded (1) A/T and C/G genotyped variants with an MAF ≥ 0.45 in 1000 Genome Project EUR subset[37]; (2) variants with a call rate <0.95 (or 0.98 call rate for variants with an MAF <0.01); (3) variants with a significant difference in genotype call rate between cases and controls (p value $< 1 \times 10^{-4}$); (4) variants with allele frequency differences versus those reported in gnomAD non-finish Europeans or TOPMed[38] global MAF (using the criterion $[(p1 - p0)^2/((p1 + p0) \times (2 - p1 - p0)] > 0.025$ and > 0.125, respectively), where p0 is the MAF in the reference panel and p1 the observed MAF in the study; (5) variants with an HWE p value $<10^{-5}$ among controls and 10^{-12} among cases; and (6) monomorphic variants. We also excluded samples with a missing genotype rate >0.5; a heterozygosity estimate +/− 4 standard deviations from the mean (per continental population), a mismatch between recorded gender and inferred genotypic sex, with a kinship coefficient ≥ 0.345 (defined using KING[39] [v2.2.4]) with another sample within the study, or ≥ 0.177 with another sample in other UK IBD study; or with evidence of non-European ancestry, defined by projecting the samples onto principal components estimated from 1000 Genomes Project reference samples.[37]

The datasets were imputed using the multi-ancestry TOPMed reference panel (r2@1.0.0) via the TOPMed imputation server[38,40] (imputationserver@1.5.7). After the first round of imputation, variants with an empirical $R^2 < 0.5$ were excluded from the analysis. Imputation was repeated after correcting strand issues at SNPs with an empirical $R^2 < -0.5$. Post imputation, variants with HWE p value $\leq 1 \times 10^{-5}$, MAF <0.001, or imputation $R^2 < 0.4$ were excluded.

The GWAS training datasets included 4,647 and 5,400 UC and CD cases, respectively, and 10,308 shared controls.

Association tests for UC and CD were performed using Regenie[41] (v1.0), including European populations principal components and sex as covariates. This produced GWAS summary statistics for 14,056,620 variants.

Building polygenic risk scores from CD and UC GWAS summary statistics

Summary statistics for CD and UC were initially filtered to remove those SNPs with an imputation INFO (or MARCH R^2) <0.8 or those failing the following quality-control thresholds:

$$\sigma_{SS} < 0.5\,\sigma_G \text{ or } \sigma_{SS} > \sigma_G + 0.1 \text{ or } \sigma_{SS} < 0.1 \text{ or } \sigma_G < 0.05,$$

where σ_{SS} is defined as

$$\sigma_{SS} = \frac{2}{\sqrt{N{\sigma_\beta}^2}},$$

and σ_G as

$$\sigma_G = \sqrt{2AF(1 - AF)},$$

where AF, N, and ${\sigma_\beta}^2$ are the minor allele frequency, the sample size, and the standard error of the SNP coefficients, respectively. The above threshold criteria used for this filtering step were sourced from the LDpred2 recommended settings. We then applied shaPRS across the remaining set of SNPs, which were then filtered to only keep SNPs that were either in the HapMap3 panel (a common practice for PRS generation) or had a trait heterogeneity lFDR <1. We chose to expand beyond HapMap3 in the case of IBD, as many of the variants that differentiated CD from UC were not captured on the standard HapMap3 panel. However, we note that shaPRS is completely agnostic to the set of SNPs it is applied to, as it can be applied to any summary data from genome-wide SNPs to the typical HapMap3-based PRS panel. To accommodate the non-HapMap3 SNPs in the LD-reference panel, we generated SNP-SNP correlation matrices for 1,703 LD blocks[42] using PLINK's "*–r*" function. The resulting files were then compressed and packaged via a custom Python script into the same *hdf5* format as used by PRS-CS. This procedure left 856,877 SNPs that were used to generate the PRS by all the

Table 1. **Cross-ancestry PRS data parameters**

Target ancestry	Trait	Adjunct study Ancestries (cases/controls or sample size)	Proximal study Ancestries (cases/controls or sample size)	SNPs in PRS
EUR	asthma	Biobank Japan,[43] East Asian (8,216/201,592)	Demenais et al.,[49] European (19,954/107,715)	752,731
	height	Biobank Japan, East Asian (159,095)	Wood et al.,[46] European (241,826)	698,742
	BRCA	Biobank Japan, East Asian (5,552/89,731)	Michailidou et al.,[50] European (14,910/17,588)	763,902
	CAD	Biobank Japan, East Asian (29,319/183,134)	Nelson et al.,[51] European (10,801/137,914)	818,926
	T2D	Biobank Japan, East Asian (36,614/155,150)	Scott et al.,[52] European (26,676/132,532)	891,047
AFR	height	Wood et al.,[46] European 241,826	Uganda Genome Resource,[45] African (14,126)	680,312
	BMI	Locke et al.,[47] European 230,965	Uganda Genome Resource, African (13,976)	638,552
	LDL	Willer et al.,[48] European (92,019)	Uganda Genome Resource, African (13,086)	660,233

evaluated methods. The final PRSs for the IBD datasets were built using PRS-CS, and the profile scores for our test set individuals were generated using PLINK's "*–score*" function.

Cross-ancestry datasets and PRS model evaluation

The Japanese association summary data for the five traits (asthma, height, breast cancer [BRCA], coronary artery disease [CAD], and type 2 diabetes [T2D]) were all retrieved from the Biobank Japan repository.[43,44] The European association data for the same five traits were sourced from different studies identified through the GWAS catalog selected based on the criteria that they were of comparable sample size and that they did not overlap with the (non-interim) UKBB release. For the scenario involving the African proximal population, we obtained African ancestry GWAS summary statistics from the Uganda Genome Resource[45] for three traits (body mass index [BMI], height, and low-density lipoprotein [LDL] cholesterol levels), with European adjunct association summary statistics from other published sources.[46–48] The full set of sources are shown in Table 1.

To produce the final LD-reference data, we used a custom R script (*LDRefGen_wrapper.R*, included in the project Git Hub) to estimate the pairwise correlations between shaPRS SNP effect estimates. This functionality is now available in our R package via the *shaPRS_LDGen* function, which requires the proximal and adjunct LD-reference panels in LDpred2 format and the shaPRS pre-processed summary data. For more information on the mathematical details of the LD derivation, see the supplemental notes. To maximize the fraction of variants available across ancestries and summary datasets, HapMap3 SNPs were chosen that were shared between the adjunct and proximal summary statistics that were also present in the UKBB imputed dataset with an INFO score >0.8. The final PRSs were built after the removal of ambiguous alleles (A/T and G/C). All PRS generation methods, LDpred2, PRS-CS, and PRS-CSx, were applied via their respective "auto" options to estimate overall shrinkage, keeping with our use case of no additional genotype data being available to fine tune hyper-parameters. PRS profiles were generated in PLINK[53] and evaluated using individual genotypes from the UKBB cohort. For all traits, we excluded related individuals and restricted the analysis to individuals of the proximal ancestry. For the generation of the proximal African (AFR) population, we identified 6,414 individuals using the population centroids published by Prive et al.,[54] and for the European (EUR) adjunct dataset, we relied on the flag White British ethnicity (UKBB field 21000, code 1001) in the UKBB documentation.[54] We also excluded ~30,000 individuals from the initial release that were genotyped with the UK BiLEVE array. We identified those individuals using the field "22000" batches coded −1 to −11. For BRCA, CAD, and T2D, we applied the same selection criteria for cases and controls as previously described,[55] using the same UKBB codes for each of the relevant traits as in https://github.com/privefl/simus-PRS/tree/master/paper3-SCT/code_real. Briefly, we included as cases those individuals who self-reported the condition or were diagnosed by a medical doctor, or the condition was included in their death record. For breast cancer, we excluded individuals with other cancer diagnoses and restricted the analysis to females (10,821 cases, 147,134 controls). For T2D we excluded individuals with type 1 diabetes (12,288 cases, 301,822 controls), and for CAD we excluded individuals with other heart conditions (10,611 cases, 209,480 controls). For the asthma phenotype, we identified individuals with the condition who had a positive response for self-reported code 20002_1111 (28,576 cases, 222,649 controls). For height, we used 251,262 individuals in total with phenotype code 50.

To quantify the performance of the PRS for binary traits we calculated the area under the curve (AUC) (for binary traits) between the predicted and observed phenotypes using the R package pROC. Similarly, for quantitative traits we calculated the squared correlation between the PRS and the measured trait (r^2). Table 1 summarizes the cross-ancestry PRS evaluation parameters.

Results

Simulations of different trait, same-ancestry datasets

We performed simulations utilizing common SNPs (MAF>1%) genotyped in the UKBB[26] cohort. We compared

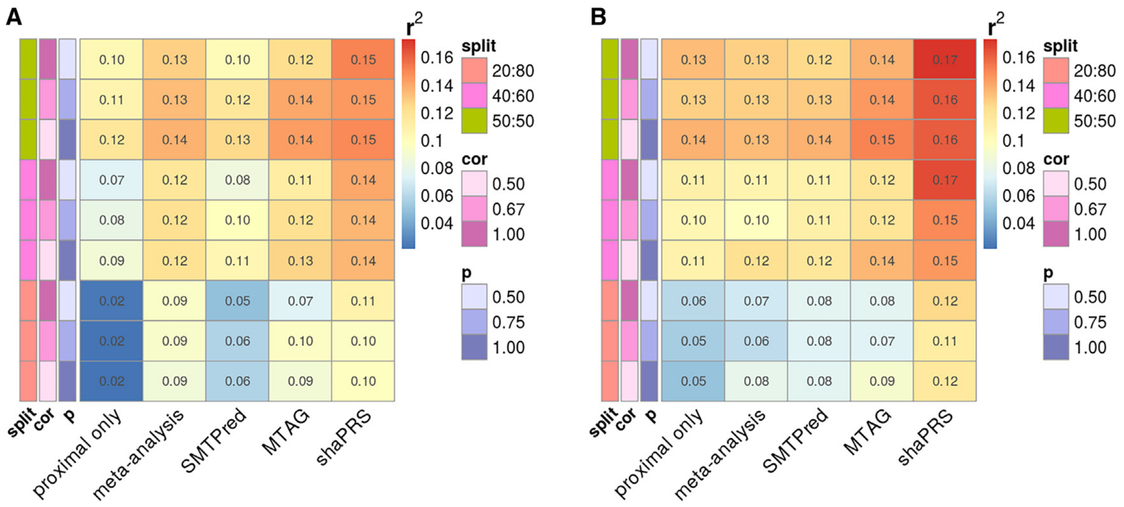

Figure 1. Heatmap of the squared correlation between simulated and predicted phenotypes for selected cross-trait genetic relationships
Warmer colors indicate better performance.
(A) A genome-wide genetic correlation between proximal and adjunct traits of 0.5 with a heritability of 0.5 from 1,000 causal variants and no extra heterogeneity created by SNPs of large effect. Sample size $n = 14{,}044$, with a proximal/adjunct sample ratio of 50/50, 40/60, or 20/80 and where cor is the correlation of effect sizes between SNPs, and P is the fraction of causal SNPs shared between the proximal and adjunct datasets. split is the ratio of the proximal to adjunct dataset sizes.
(B) The same scenario as (A), with the addition of extra heterogeneity created by five SNPs of large effect that contributed 5% non-shared heritability.
Results across the complete set of simulated scenarios are shown in Figure S2.

shaPRS to two baseline approaches: single-dataset analysis (β_1 at all SNPs) and inverse variance weighted meta-analysis (β_{12} at all SNPs). The meta-analysis is equivalent to running shaPRS if there was no heterogeneity of effect anywhere across the genome, allowing us to quantify the extent to which incorporating the measure of heterogeneity (lFDR) learned via the Cochran test improves PRSs. In recent years, several methods that exploit the genetic correlation between related traits to improve association or prediction accuracies have been proposed, including SMTPred,[20] MTAG,[19] and CTPR.[56] We choose SMTPred and MTAG as comparison methods because they also rely on genome-wide summary statistics and thus have a similar use case to shaPRS. However, like other previously developed methods, both SMTPred and MTAG assume a constant shared genetic etiology across the genome. A detailed description of the simulation can be found in the material and methods section.

Genetic correlation (rG), which is a scalar metric, does not fully capture the overall structure of shared genetic etiology. For example, a genetic correlation of 0.5 can be the result of all causal SNPs shared with a per-SNP effect correlation of 0.5, or only half of the causal SNPs may be shared but with an effect correlation of 1.0. By fixing the genetic correlation at 0.5 but varying the fraction of shared and non-shared genetic effects, we investigated and demonstrated the key ability of our method to adapt to such different compositions of overlapping genetic etiologies. We also considered an additional scenario, where five SNPs contribute 5% of the total non-shared heritability for each trait. The rationale for including such SNPs was to model highly penetrant variants in genes such as *NOD2* in CD[25] or *FLT3* in autoimmune thyroid disease,[24,57] which play an important role in differentiating these diseases from otherwise genetically related conditions. Our main simulation analyses examined 162 different genetic architectures that arose from the examined parameters. The full set of parameters are summarized in Table S1, and Figure 1 presents a subset of our simulation results with a heritability of 0.5, 1,000 causal variants, and an rG of 0.5 between the proximal and adjunct datasets. The full set of results from all simulation scenarios can be found in Figures S2 and S3.

shaPRS outperformed alternative methods in 93% of the simulated scenarios and did so frequently by large margins. Figure S4 visualizes the formal evaluation (via *r2redux*' *r2_diff* function) between shaPRS and other methods as a heatmap for the scenarios depicted in Figure 1. ShaPRS' capacity to accommodate genetic heterogeneity at a per-SNP level was particularly demonstrated by an increasingly larger performance advantage over other methods in scenarios (Figure S1) where a given genetic correlation between two traits was concentrated among a subset of causal SNPs with stronger effect size correlations (see rG composition in Table S1). Reassuringly, shaPRS performed similarly to other methods in scenarios with a constant shared genetic etiology (all causal SNPs shared between traits with weaker correlation in effect sizes). The relative ordering of the performance of the methods did not change with the introduction of extra heterogeneity created by SNPs of large effect (Figures 1B and S1B). However, such high penetrance variants further enhanced the advantage of shaPRS against all evaluated alternatives. In conclusion, shaPRS compared favorably to all other approaches, particularly in scenarios when the underlying assumption of no non-shared SNPs with non-null effects was violated.

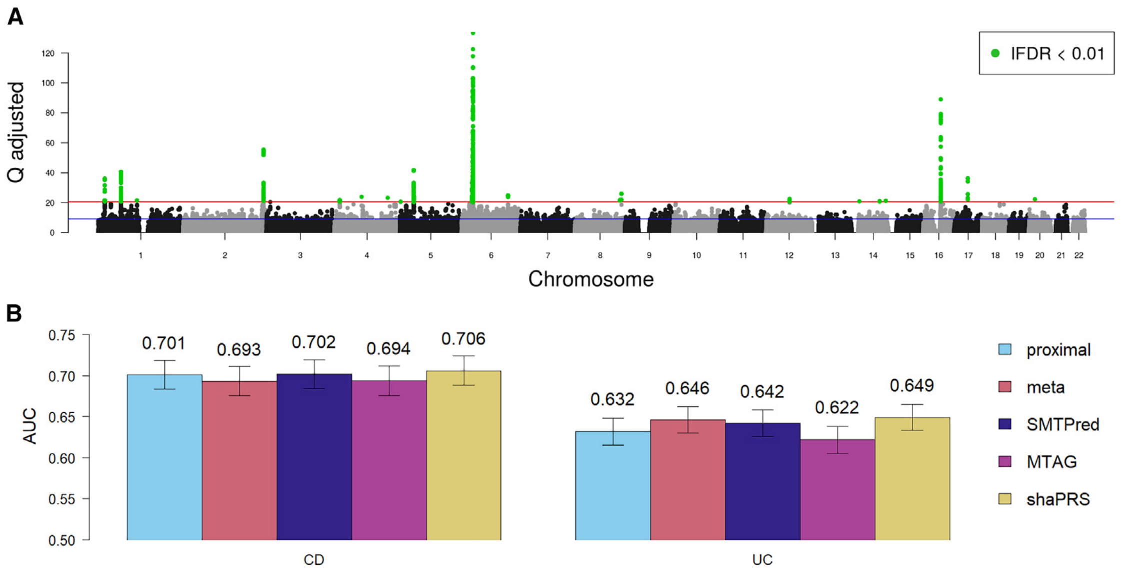

Figure 2. Comparison of PRS estimation methods for predicting IBD subtypes

(A) Manhattan plot depicting the genome-wide heterogeneity between Crohn disease and ulcerative colitis measured by Cochran's Q test (y axis). Blue line represents SNPs with an lFDR <0.5, and the red line represents SNPs with an lFDR <0.01, which are also highlighted in green.

(B) Barplot of PRS performance evaluated by the area under the receiver operating characteristic curve of the predicted and observed phenotypes in independent cohorts of 1,181/2,896 and 1,909/2,764 cases/controls, for Crohn disease (CD) and ulcerative colitis (UC), respectively. Each colored bar represents a different PRS estimation method: IBD subtype alone (proximal: cyan), fixed-effect meta-analysis (meta: orange), SMTPred (dark blue), MTAG (pink), and shaPRS (yellow). The error bars represent the 95% confidence intervals, which were computed with 2,000 stratified bootstrap replicates, and the values above each bar show the AUC for the given method.

Application to inflammatory bowel disease subtypes

Inflammatory bowel disease (IBD) is a complex inflammatory disease of the gastrointestinal tract with a prevalence of 0.5% in Western countries.[58] Its two main clinical subtypes, CD and UC have a substantial but imperfect overlap in their genetic etiologies, with a genome-wide genetic correlation of ~0.56.[59] We performed a shaPRS analysis of UC and CD using a GWAS dataset[34] that included 4,647 and 5,400 UC and CD cases, respectively, and 10,308 shared controls. The Manhattan plot in Figure 2A illustrates how the estimated lFDR values capture the landscape of heterogeneity between UC and CD with areas of highly incongruent effects (such as the *NOD2* region on chromosome 16) featuring prominently among the peaks.

A set of three baseline PRSs was built, trained either on summary statistics from a case/control GWAS of a single disease subtype (CD or UC alone) or alternatively from a fixed-effect meta-analysis of the CD and UC GWAS summary statistics to create a GWAS for the IBD phenotype). Three additional PRSs were built based on more advanced models implemented in SMTPred, MTAG, and shaPRS. All PRS were built using PRS-CS. We evaluated PRS performance on independent CD[26,36] and UC[35] cohorts, with 1,181/2,896 and 1,909/2,764 cases/controls, respectively, by estimating the squared correlation between the predicted and observed phenotypes (Figure 2; Table S3).

We found that the PRSs estimated from either the GWAS of CD or UC predicted the corresponding subtype with similar accuracy to the PRSs generated from the IBD fixed-effect meta-analysis. Considering the variance-bias trade-off latent in these experiments, these results make intuitive sense; we approximately doubled the sample size of the cases for traits that share approximately half their genetic etiology ($rG = 0.56$). Therefore, given this level of shared genetic etiology, combining phenotypes to train PRSs did not consistently improve the accuracy. However, we found that shaPRS substantially outperformed these baseline PRSs. Evaluated against the proximal dataset alone, shaPRS improved results by ~4% and ~22% for CD and UC, respectively. Compared to combining the CD and UC phenotypes in a fixed-effect meta-analysis, shaPRS increased performance by ~12% and ~6% for CD and UC, respectively. Additionally, shaPRS also outperformed SMTPred by ~7% and ~10% and MTAG by ~11% and 39% for CD and UC, respectively. ShaPRS was significantly better than both SMTPred and MTAG, by at least either the *r2redux r2_var* or the Delong tests (Table S4). We also found that adding shaPRS into a nested model of the other method always improved the overall model fit, whereas the other way around, adding the other method into a nested shaPRS-only model only improved the model fit once (in the case of MTAG for CD).

Leveraging datasets from different ancestries

To date, GWASs have been predominantly focused on European populations. The accuracy of PRSs generated from GWAS summary statistics in one ancestry is decreased in individuals of other ancestries due to a combination of differences in LD, MAF, and causal variant effects between

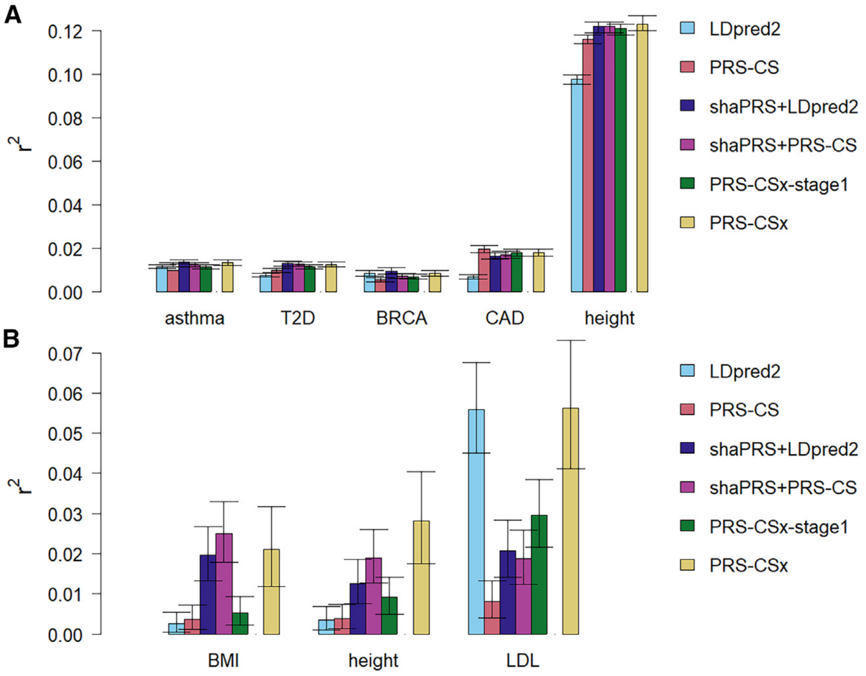

Figure 3. shaPRS maximizes the accuracy of PRSs across divergent ancestry groups when only GWAS summary statistics are available

LDpred2 and PRS-CS trained on only the proximal ancestry datasets. shaPRS + LDpred2 and shaPRS + PRS-CS add preprocessing by shaPRS to leverage the adjunct datasets while generating a proximal ancestry PRS. PRS-CSx-stage1 combines the proximal and adjunct summary data without the reliance on additional genotype validation data. PRS-CSx follows on from PRS-CSx-stage1 by performing an additional step of finding the best linear combination of the proximal and adjunct PRS files by using additional genotype validation data. As PRS-CSx assumes a different use case than our paper, it is only included as a point of reference.

(A) Barplot of PRS performance for the EUR proximal and EAS adjunct analyses, evaluated by the squared Pearson correlation coefficient (r^2) between predicted and observed phenotypes. Confidence intervals were generated via the *r2redux*' *r2_var* function. T2D, type 2 diabetes; BRCA, breast cancer; CAD, coronary artery disease.

(B) Barplot of PRS performance for the AFR proximal and EUR adjunct analyses, evaluated by the squared Pearson correlation coefficient (r^2) between predicted and observed phenotypes. BMI, body mass index; LDL, low-density lipoprotein cholesterol level.

populations. We hypothesized that shaPRS could better leverage information from GWASs in different ancestries to construct more performant PRSs. To test this hypothesis, we obtained GWAS summary statistics across a range of traits from diverse population pairs (Table 1, material and methods) and quantified the extent to which shaPRS' use of the adjunct data improved the performance of two PRS methods (PRS-CS and LDpred2) that only make use of GWAS summary statistics from the proximal ancestry. These methods require LD matrices representative of the study population and are therefore restricted to single population analysis. We present their standard results as baselines, as well as the results with shaPRS pre-processing to leverage information from the adjunct datasets. Both methods can either use an additional validation dataset to optimize parameters or estimate these internally using an auto mode. We used the auto mode to focus on the situation where an independent set of summary statistics is unavailable for the proximal population (which we believe will predominantly be the case for understudied populations). We also compared shaPRS against PRS-CSx,[23] a recently developed method that integrates GWAS summary statistics across different ancestries while accounting for MAF and LD differences. PRS-CSx is performed in two stages. Stage 1 infers posterior SNP effect sizes under continuous shrinkage (CS) priors, learnt either directly from the proximal dataset (auto mode) or optimized using a second independent set of GWAS summary statistics from the same proximal population. We again chose to use the auto approach and refer to this as PRS-CSx-stage1. Note that this is not the recommended way to run PRS-CSx, and we include stage1 only to assess the performance without any additional dataset. Finally, to quantify the added value an independent proximal dataset brings to PRS performance, over and above any provided by shaPRS, we provided PRS-CSx with an independent dataset over which to optimize the weighted averaging of effects between the proximal and adjunct ancestries, which we refer to as PRS-CSx. The performance of each PRS method was evaluated by estimating the r^2 and AUC (for binary traits) between the predicted and observed phenotypes (Figure 3; Table S2). We have also performed formal significance tests and likelihood-ratio tests that compared shaPRS and the other method baselines, relative to a complex model that had both (Table S5).

ShaPRS improved the accuracy of PRS estimates from LDpred2 and PRS-CS in six of the eight traits studied, with the greatest improvement seen for traits where the power of the adjunct dataset far outweighed that of the proximal study (e.g., BMI and height in the $\text{African}_{\text{proximal}}$ and $\text{European}_{\text{adjunct}}$ studies). shaPRS also consistently outperformed PRS-CSx-stage1 in these same six studies, with four instances also reaching statistical significance (Table S5), demonstrating its superiority in situations where only a single set of GWAS summary statistics are available for a given proximal population. Furthermore, the shaPRS improved PRSs were more performant than those from PRS-CSx for four of the eight traits studied, despite the fact that PRS-CSx exclusively made use of an independent dataset from the proximal population. PRS-CSx only outperformed shaPRS improved scores by a noticeable margin for two of the eight tested traits (height and LDL in the $\text{African}_{\text{proximal}}$ and $\text{European}_{\text{adjunct}}$ studies), highlighting the extent to which shaPRS can improve PRS without the need for a second independent set of GWAS summary statistics from the proximal population.

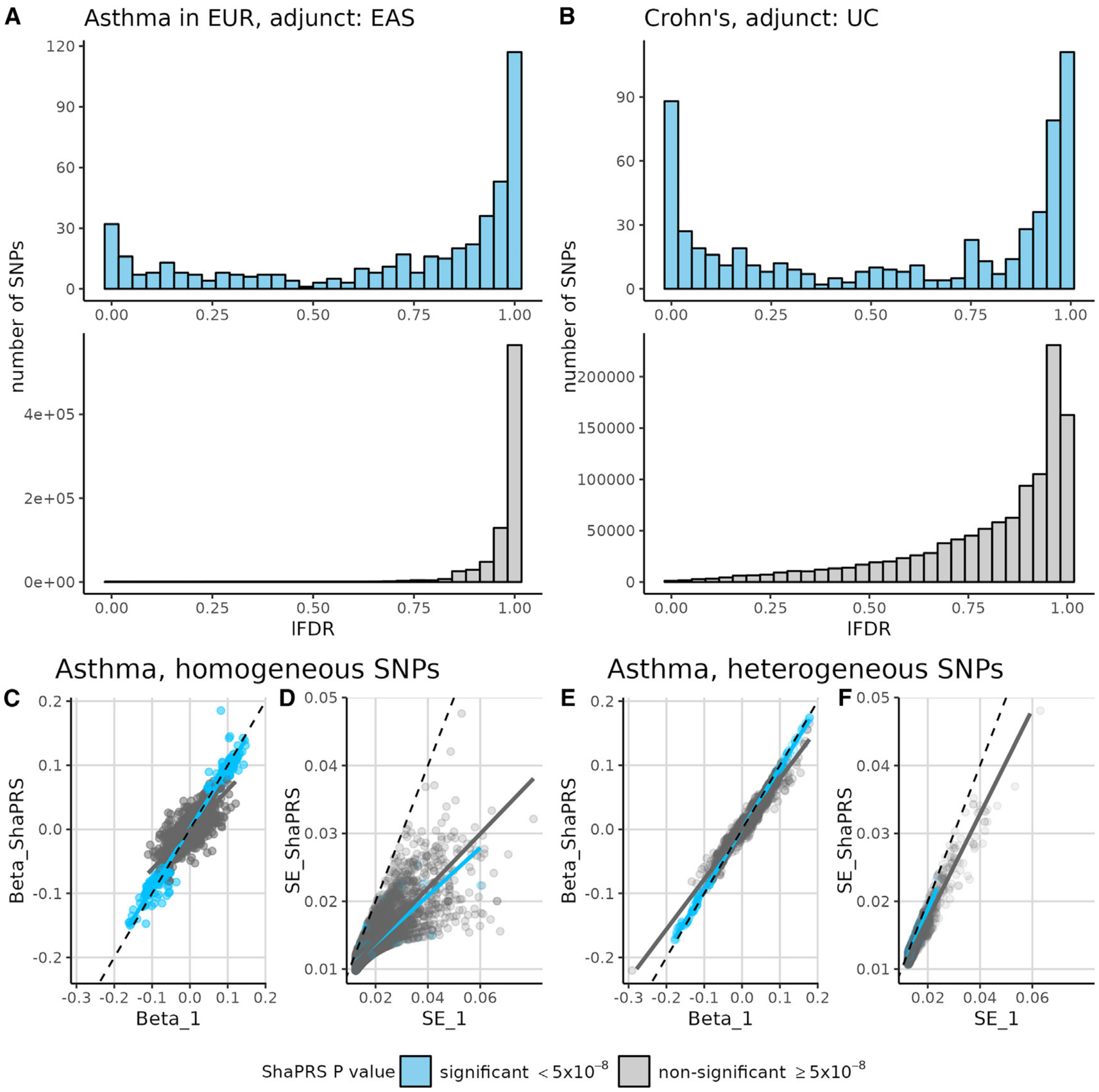

Figure 4. shaPRS maximizes accuracy of PRSs by more completely capturing between SNP variation in shared effects across ancestry groups or related traits

The top row contrasts the distribution of effect heterogeneity measured by lFDR in (A) a cross-ancestry analysis of asthma that uses European GWAS summary statistics as the proximal dataset and East Asian GWAS summary statistics as the adjunct dataset (left) and (B) a cross-trait analysis of Crohn disease (CD), leveraging a GWAS of ulcerative colitis (UC) as an adjunct dataset. The distributions of lFDR values are shown, where low lFDR corresponds to higher heterogeneity in estimated effects. The bottom row compares the proximal study's beta (Beta_1) and standard error (SE_1) to its shaPRS-adjusted output (Beta_shaPRS and SE_shaPRS, respectively) for the asthma analysis. SNPs have been partitioned into those with little evidence of heterogeneity (lFDR >0.5) between the two populations (C and D) and this with evidence of heterogeneity (lFDR ≤ 0.5) between the two populations (E and F). Colors indicate whether a SNP was detected to have a significantly non-zero effect ($p < 5 \times 10^{-8}$) in the shaPRS analysis.

To better understand how shaPRS gains performance, we examined two analyses in detail. Taking the asthma analysis in EUR/EAS individuals and the CD/UC analysis as examples, we can see that very few SNPs are detected to have genuinely different effects (i.e., low lFDR) in the different ancestries or traits (Figures 4A and 4B). Among SNPs with low heterogeneity (Figure 4C), shaPRS tends to shrink the effect size, beta, toward zero for SNPs ultimately declared non-significant while leaving beta on average unchanged for significant SNPs. For all low heterogeneity SNPs, the standard error shrinks as would be expected in any meta-analysis. For high heterogeneity SNPs (Figure 4D), on the other hand, both beta and the standard error remain relatively unchanged regardless of significance. shaPRS thus allows effects specific to individual traits or populations to be leveraged when appropriate and does not attempt to leverage that information when inappropriate. Given the predominance of low heterogeneity, non-significant SNPs, the greatest effect of shaPRS is to shrink estimates of null SNPs toward zero.

Discussion

We introduce shaPRS, a method that integrates genetic association information from heterogeneous sources to

improve the accuracy of PRS for related traits and across ancestral populations.

A major strength of shaPRS is the ability to exploit the differential genetic architecture of related traits by considering the evidence for heterogeneity at each variant and weighting toward the estimate with the more beneficial properties: smaller variance in case of low heterogeneity or, alternatively, smaller bias in case of high heterogeneity. shaPRS can thus particularly improve the accuracy of a PRS when the genetic correlation structure between the proximal and adjunct datasets varies between SNPs. This is in contrast with previously developed methods, such as SMTPred and MTAG, both of which assume a constant sharing of genetic etiology. SMTPred assumes that all SNP effects are shared equally (it integrates predictions on the PRS-level) whereas MTAG learns different per-SNP weights; however, unlike shaPRS, the combined SNP effects are a function of parameters that are constant across all variants. The per-SNP weighting approach adopted by shaPRS requires a given SNP to be genotyped in both the proximal and adjunct studies, otherwise the effect from the proximal study alone is used to generate the PRS. The advantage of applying shaPRS pre-processing is therefore positively correlated with the number of SNPs genotyped in both the proximal and adjunct datasets. Given the widespread use of a small number of genotyping arrays and genotype imputation, we expect most traits or ancestries to have sufficient overlapping SNPs to make shaPRS pre-processing a useful strategy.

In our example of CD and ulcerative colitis, the pervasive sharing of genetic effects between the two diseases is well established,[60] and the genetic correlation between the two diseases has been estimated to be 0.56[55]. However, there are some SNPs with large differences in effect between CD and UC[60]; for example, in the *NOD2* locus genetic variants explain around 1.5% of the variance in liability of CD,[61] but there is no evidence of association to UC. More fully accounting for this inconsistent correlation in genetic effects between traits enables shaPRS to outperform competing cross-trait methods. In the case of CD risk prediction, shaPRS outperformed fixed-effect meta-analysis, SMTPred, and MTAG by ∼12%, ∼7%, and ∼11%, respectively (see Table S3 for detailed results).

When applying our method to cross-ancestry prediction based solely on GWAS summary statistics, shaPRS with either LDpred2 or PRS-CS, outperformed the cross-ancestry method PRS-CSx for six of the eight traits considered. Even when we exclusively provided PRS-CSx with this additional genotyped dataset to fine-tune the final PRS by finding the best linear combination of the proximal and adjunct PRS, it only appreciably outperformed a shaPRS-informed analysis for two of the eight tested traits. We note that such a fine-tuning approach could, in principle, be applied to the outputs of any PRS method (including those built by shaPRS) to further improve their PRS accuracy across populations. Unfortunately, we believe that additional genotyped datasets will seldom be available for most underrepresented populations and lower prevalence traits, making shaPRS' ability to generate accurate PRSs without these a key feature of our method.

Comparing the full with the nested models of either just shaPRS or just one of the other methods also demonstrated our method's advantages, as evidenced by the consistently lower likelihood-ratio test p values favoring shaPRS. This held true for both the simulations (Figure S5), as well as for the majority of the real data analyses (Tables S4 and S5), despite the limited sample sizes and therefore lower power for the latter. We interpret these results to suggest that shaPRS was able to achieve higher performance by adding unique information not available to the other methods.

In the coming years, to expand the clinical applicability of PRS, more ancestrally diverse populations will need to be recruited for large-scale genetic research.[12,13] In the interim, methods such as shaPRS can contribute to more equitable health outcomes by leveraging existing datasets more effectively. Our simulations and real-world examples show that shaPRS can improve PRS estimation across a broad range of genetic architectures. While we have showcased the power of shaPRS for improving PRS estimates between traits and ancestries, this flexibility enables shaPRS to be applied whenever incomplete sharing of genetic effects is expected between two GWAS datasets. Other possible use cases for shaPRS could therefore include generating PRS for traits with heterogeneity of effect between the sexes or between different environments.

shaPRS is designed to fit within existing pipelines as a pre-processing tool, thus, it is not in direct competition with other PRS generation tools such as LDpred2[29] or PRS-CS.[30] shaPRS can therefore continue to be applied as more performant PRS methods are derived in the future, such as the recently proposed PolyPred.[62] Our recommended approach is to pre-process GWAS summary statistics via shaPRS before taking them forward to a PRS tool of choice that would be used to produce the final profile scores. shaPRS also fits with the ongoing trend of reliance on summary statistics alone, without the need for access to genotype-level data at any stage, as it provides a competitive performance without the need for a validation genotype cohort. Our method is open source and is freely available from https://github.com/mkelcb/shaprs.

Adjunct study is the source of the summary statistics for the adjunct ancestry data (Japanese for the European target PRS and European for the African target PRS). Proximal study is the source of the summary statistics for the target ancestry data. SNPs in PRS are the number of SNPs in the polygenic score for all methods. Data on coronary artery disease/myocardial infarction have been contributed by the CARDIoGRAMplusC4D and UKBB CardioMetabolic Consortium CHD working group who used the UKBB Resource (application number 9922). Data have been downloaded from www.cardiogramplusc4d.org. For the European datasets, for the traits CAD, height, BMI, and LDL, the per-SNP sample sizes differed, thus the values shown represent the mean.

Data and code availability

shaPRS R package is available from https://github.com/mkelcb/shaprs. Code to perform all analyses reported in this manuscript is available at https://github.com/mkelcb/shaprs-paper. The final PRS files and diagnostic data are available from the supplemental information.

Supplemental information

Supplemental information can be found online at https://doi.org/10.1016/j.ajhg.2024.04.009.

Acknowledgments

This work was funded by the Wellcome Trust (220540/Z/20/A, Wellcome Sanger Institute Quinquennial Review 2021–2026, 203950/Z/16/A, WT220788, WT107881, 206194, and 108413/A/15/D) and the MRC (MC_UU_00002/4) and supported by the NIHR Cambridge BRC (BRC-1215-20014). The views expressed are those of the author(s) and not necessarily those of the NHS, the NIHR or the Department of Health and Social Care. For the purpose of Open Access, the author has applied a CC BY public copyright license to any Author Accepted Manuscript version arising from this submission.

This research was conducted using the UK Biobank Resource under application number 30931.

We thank all individuals who donated or collected samples used in this study.

Declaration of interests

C.A.A. has received consultancy or lectureship fees from Genomics plc, BridgeBio, Inc., and GSK. C.W. receives funding from GSK and MSD and is a part time employee of GSK. These companies had no input on this work.

Received: July 28, 2023
Accepted: April 15, 2024
Published: May 3, 2024

Web resources

European Genome-Phenome Archive, Genotyping of additional Inflammatory Bowel Disease cases, https://ega-archive.org/studies/EGAS00001000924,

European Genome-Phenome Archive, WTCCC2 case-control study for Ulcerative Colitis,https://ega-archive.org/studies/EGAS00000000084

European Genome-Phenome Archive, WTCCC1 project Inflammatory Bowel Disease (IBD) samples, https://ega-archive.org/datasets/EGAD00000000005

European LD reference (with blocks), https://figshare.com/articles/dataset/European_LD_reference_with_blocks_/19213299

Index of /hhuang/public/PRS-CSx/Reference, https://personal.broadinstitute.org/hhuang//public//PRS-CSx/Reference/, respectively.

References

1. Khera, A.V., Chaffin, M., Aragam, K.G., Haas, M.E., Roselli, C., Choi, S.H., Natarajan, P., Lander, E.S., Lubitz, S.A., Ellinor, P.T., and Kathiresan, S. (2018). Genome-wide polygenic scores for common diseases identify individuals with risk equivalent to monogenic mutations. Nat. Genet. *50*, 1219–1224.
2. Inouye, M., Abraham, G., Nelson, C.P., Wood, A.M., Sweeting, M.J., Dudbridge, F., Lai, F.Y., Kaptoge, S., Brozynska, M., Wang, T., et al. (2018). Genomic Risk Prediction of Coronary Artery Disease in 480,000 Adults: Implications for Primary Prevention. J. Am. Coll. Cardiol. *72*, 1883–1893.
3. McCarthy, M., and Birney, E. (2021). Personalized profiles for disease risk must capture all facets of health. Nature *597*, 175–177.
4. Mars, N., Koskela, J.T., Ripatti, P., Kiiskinen, T.T.J., Havulinna, A.S., Lindbohm, J.V., Ahola-Olli, A., Kurki, M., Karjalainen, J., Palta, P., et al. (2020). Polygenic and clinical risk scores and their impact on age at onset and prediction of cardiometabolic diseases and common cancers. Nat. Med. *26*, 549–557.
5. Zhang, Y., Qi, G., Park, J.-H., and Chatterjee, N. (2018). Estimation of complex effect-size distributions using summary-level statistics from genome-wide association studies across 32 complex traits. Nat. Genet. *50*, 1318–1326.
6. O'Connor, L.J. (2021). The distribution of common-variant effect sizes. Nat. Genet. *53*, 1243–1249.
7. Popejoy, A.B., and Fullerton, S.M. (2016). Genomics is failing on diversity. Nature *538*, 161–164.
8. Mills, M.C., and Rahal, C. (2020). The GWAS Diversity Monitor tracks diversity by disease in real time. Nat. Genet. *52*, 242–243.
9. Cavazos, T.B., and Witte, J.S. (2021). Inclusion of variants discovered from diverse populations improves polygenic risk score transferability. HGG Adv. *2*, 100017.
10. Kim, M.S., Patel, K.P., Teng, A.K., Berens, A.J., and Lachance, J. (2018). Genetic disease risks can be misestimated across global populations. Genome Biol. *19*, 179.
11. Ishigaki, K., Akiyama, M., Kanai, M., Takahashi, A., Kawakami, E., Sugishita, H., Sakaue, S., Matoba, N., Low, S.K., Okada, Y., et al. (2020). Large-scale genome-wide association study in a Japanese population identifies novel susceptibility loci across different diseases. Nat. Genet. *52*, 669–679.
12. Sirugo, G., Williams, S.M., and Tishkoff, S.A. (2019). The Missing Diversity in Human Genetic Studies. Cell *177*, 1080.
13. Rotimi, C.N., and Adeyemo, A.A. (2021). From one human genome to a complex tapestry of ancestry. Nature *590*, 220–221.
14. Zhou, W., Kanai, M., Wu, K.H.H., Rasheed, H., Tsuo, K., Hirbo, J.B., Wang, Y., Bhattacharya, A., Zhao, H., Namba, S., et al. (2022). Global Biobank Meta-analysis Initiative: Powering genetic discovery across human disease. Cell Genom. *2*, 100192.
15. Bentley, A.R., Chen, G., Doumatey, A.P., Shriner, D., Meeks, K.A.C., Gouveia, M.H., Ekoru, K., Zhou, J., Adeyemo, A., and Rotimi, C.N. (2021). GWAS in Africans identifies novel lipids loci and demonstrates heterogenous association within Africa. Hum. Mol. Genet. *30*, 2205–2214.
16. Adeyemo, A.A., Zaghloul, N.A., Chen, G., Doumatey, A.P., Leitch, C.C., Hostelley, T.L., Nesmith, J.E., Zhou, J., Bentley, A.R., Shriner, D., et al. (2019). ZRANB3 is an African-specific type 2 diabetes locus associated with beta-cell mass and insulin response. Nat. Commun. *10*, 3195.
17. Kuchenbaecker, K., Telkar, N., Reiker, T., Walters, R.G., Lin, K., Eriksson, A., Gurdasani, D., Gilly, A., Southam, L., Tsafantakis, E., et al. (2019). The transferability of lipid loci across African, Asian and European cohorts. Nat. Commun. *10*, 4330.

18. Liu, J.Z., van Sommeren, S., Huang, H., Ng, S.C., Alberts, R., Takahashi, A., Ripke, S., Lee, J.C., Jostins, L., Shah, T., et al. (2015). Association analyses identify 38 susceptibility loci for inflammatory bowel disease and highlight shared genetic risk across populations. Nat. Genet. *47*, 979–986.
19. Turley, P., Walters, R.K., Maghzian, O., Okbay, A., Lee, J.J., Fontana, M.A., Nguyen-Viet, T.A., Wedow, R., Zacher, M., Furlotte, N.A., et al. (2018). Multi-trait analysis of genome-wide association summary statistics using MTAG. Nat. Genet. *50*, 229–237.
20. Maier, R.M., Zhu, Z., Lee, S.H., Trzaskowski, M., Ruderfer, D.M., Stahl, E.A., Ripke, S., Wray, N.R., Yang, J., Visscher, P.M., and Robinson, M.R. (2018). Improving genetic prediction by leveraging genetic correlations among human diseases and traits. Nat. Commun. *9*, 989.
21. Márquez-Luna, C., Loh, P.-R., South Asian Type 2 Diabetes SAT2D Consortium; and SIGMA Type 2 Diabetes Consortium, and Price, A.L. (2017). Multiethnic polygenic risk scores improve risk prediction in diverse populations. Genet. Epidemiol. *41*, 811–823.
22. Marnetto, D., Pärna, K., Läll, K., Molinaro, L., Montinaro, F., Haller, T., Metspalu, M., Mägi, R., Fischer, K., and Pagani, L. (2020). Ancestry deconvolution and partial polygenic score can improve susceptibility predictions in recently admixed individuals. Nat. Commun. *11*, 1628.
23. Ruan, Y., Lin, Y.F., Feng, Y.C.A., Chen, C.Y., Lam, M., Guo, Z., Stanley Global Asia Initiatives, He, L., Sawa, A., Martin, A.R., et al. (2022). Improving polygenic prediction in ancestrally diverse populations. Nat. Genet. *54*, 573–580.
24. Cooper, J.D., Simmonds, M.J., Walker, N.M., Burren, O., Brand, O.J., Guo, H., Wallace, C., Stevens, H., Coleman, G.; and Wellcome Trust Case Control Consortium (2012). Seven newly identified loci for autoimmune thyroid disease. Hum. Mol. Genet. *21*, 5202–5208.
25. Waterman, M., Xu, W., Stempak, J.M., Milgrom, R., Bernstein, C.N., Griffiths, A.M., Greenberg, G.R., Steinhart, A.H., and Silverberg, M.S. (2011). Distinct and overlapping genetic loci in Crohn's disease and ulcerative colitis: correlations with pathogenesis. Inflamm. Bowel Dis. *17*, 1936–1942.
26. Sudlow, C., Gallacher, J., Allen, N., Beral, V., Burton, P., Danesh, J., Downey, P., Elliott, P., Green, J., Landray, M., et al. (2015). UK biobank: an open access resource for identifying the causes of a wide range of complex diseases of middle and old age. PLoS Med. *12*, e1001779.
27. Storey, J.D. (2011). False Discovery Rate. In International Encyclopedia of Statistical Science (Springer Berlin), pp. 504–508.
28. Privé, F., Arbel, J., Aschard, H., and Vilhjálmsson, B.J. (2022). Identifying and correcting for misspecifications in GWAS summary statistics and polygenic scores. HGG Adv. *3*, 100136.
29. Privé, F., Arbel, J., and Vilhjálmsson, B.J. (2021). LDpred2: better, faster, stronger. Bioinformatics *36*, 5424–5431.
30. Ge, T., Chen, C.-Y., Ni, Y., Feng, Y.-C.A., and Smoller, J.W. (2019). Polygenic prediction via Bayesian regression and continuous shrinkage priors. Nat. Commun. *10*, 1776.
31. Lin, D.-Y., and Sullivan, P.F. (2009). Meta-analysis of genome-wide association studies with overlapping subjects. Am. J. Hum. Genet. *85*, 862–872.
32. Speed, D., Holmes, J., and Balding, D.J. (2020). Evaluating and improving heritability models using summary statistics. Nat. Genet. *52*, 458–462.
33. Reales, G., Vigorito, E., Kelemen, M., and Wallace, C. (2021). RápidoPGS: a rapid polygenic score calculator for summary GWAS data without a test dataset. Bioinformatics *37*, 4444–4450.
34. de Lange, K.M., Moutsianas, L., Lee, J.C., Lamb, C.A., Luo, Y., Kennedy, N.A., Jostins, L., Rice, D.L., Gutierrez-Achury, J., Ji, S.G., et al. (2017). Genome-wide association study implicates immune activation of multiple integrin genes in inflammatory bowel disease. Nat. Genet. *49*, 256–261.
35. UK IBD Genetics Consortium, Barrett, J.C., Lee, J.C., Lees, C.W., Prescott, N.J., Anderson, C.A., Phillips, A., Wesley, E., Parnell, K., Zhang, H., et al. (2009). Genome-wide association study of ulcerative colitis identifies three new susceptibility loci, including the HNF4A region. Nat. Genet. *41*, 1330–1334.
36. Wellcome Trust Case Control Consortium (2007). Genome-wide association study of 14,000 cases of seven common diseases and 3,000 shared controls. Nature *447*, 661–678.
37. 1000 Genomes Project Consortium, Auton, A., Brooks, L.D., Durbin, R.M., Garrison, E.P., Kang, H.M., Korbel, J.O., Marchini, J.L., McCarthy, S., McVean, G.A., and Abecasis, G.R. (2015). A global reference for human genetic variation. Nature *526*, 68–74.
38. Taliun, D., Harris, D.N., Kessler, M.D., Carlson, J., Szpiech, Z.A., Torres, R., Taliun, S.A.G., Corvelo, A., Gogarten, S.M., Kang, H.M., et al. (2021). Sequencing of 53,831 diverse genomes from the NHLBI TOPMed Program. Nature *590*, 290–299.
39. Manichaikul, A., Mychaleckyj, J.C., Rich, S.S., Daly, K., Sale, M., and Chen, W.M. (2010). Robust relationship inference in genome-wide association studies. Bioinformatics *26*, 2867–2873.
40. Das, S., Forer, L., Schönherr, S., Sidore, C., Locke, A.E., Kwong, A., Vrieze, S.I., Chew, E.Y., Levy, S., McGue, M., et al. (2016). Next-generation genotype imputation service and methods. Nat. Genet. *48*, 1284–1287.
41. Mbatchou, J., Barnard, L., Backman, J., Marcketta, A., Kosmicki, J.A., Ziyatdinov, A., Benner, C., O'Dushlaine, C., Barber, M., Boutkov, B., et al. (2021). Computationally efficient whole-genome regression for quantitative and binary traits. Nat. Genet. *53*, 1097–1103.
42. Berisa, T., and Pickrell, J.K. (2016). Approximately independent linkage disequilibrium blocks in human populations. Bioinformatics *32*, 283–285.
43. Nagai, A., Hirata, M., Kamatani, Y., Muto, K., Matsuda, K., Kiyohara, Y., Ninomiya, T., Tamakoshi, A., Yamagata, Z., Mushiroda, T., et al. (2017). Overview of the BioBank Japan Project: Study design and profile. J. Epidemiol. *27*, S2–S8.
44. Sakaue, S., Kanai, M., Tanigawa, Y., Karjalainen, J., Kurki, M., Koshiba, S., Narita, A., Konuma, T., Yamamoto, K., Akiyama, M., et al. (2021). A cross-population atlas of genetic associations for 220 human phenotypes. Nat. Genet. *53*, 1415–1424.
45. Gurdasani, D., Carstensen, T., Fatumo, S., Chen, G., Franklin, C.S., Prado-Martinez, J., Bouman, H., Abascal, F., Haber, M., Tachmazidou, I., et al. (2019). Uganda Genome Resource Enables Insights into Population History and Genomic Discovery in Africa. Cell *179*, 984–1002.e36.
46. Wood, A.R., Esko, T., Yang, J., Vedantam, S., Pers, T.H., Gustafsson, S., Chu, A.Y., Estrada, K., Luan, J., Kutalik, Z., et al. (2014). Defining the role of common variation in the genomic and biological architecture of adult human height. Nat. Genet. *46*, 1173–1186.
47. Locke, A.E., Kahali, B., Berndt, S.I., Justice, A.E., Pers, T.H., Day, F.R., Powell, C., Vedantam, S., Buchkovich, M.L., Yang, J., et al.

(2015). Genetic studies of body mass index yield new insights for obesity biology. Nature *518*, 197–206.

48. Willer, C.J., Schmidt, E.M., Sengupta, S., Peloso, G.M., Gustafsson, S., Kanoni, S., Ganna, A., Chen, J., Buchkovich, M.L., Mora, S., et al. (2013). Discovery and refinement of loci associated with lipid levels. Nat. Genet. *45*, 1274–1283.
49. Demenais, F., Margaritte-Jeannin, P., Barnes, K.C., Cookson, W.O.C., Altmüller, J., Ang, W., Barr, R.G., Beaty, T.H., Becker, A.B., Beilby, J., et al. (2018). Multiancestry association study identifies new asthma risk loci that colocalize with immune-cell enhancer marks. Nat. Genet. *50*, 42–53.
50. Michailidou, K., Lindström, S., Dennis, J., Beesley, J., Hui, S., Kar, S., Lemaçon, A., Soucy, P., Glubb, D., Rostamianfar, A., et al. (2017). Association analysis identifies 65 new breast cancer risk loci. Nature *551*, 92–94.
51. Nelson, C.P., Goel, A., Butterworth, A.S., Kanoni, S., Webb, T.R., Marouli, E., Zeng, L., Ntalla, I., Lai, F.Y., Hopewell, J.C., et al. (2017). Association analyses based on false discovery rate implicate new loci for coronary artery disease. Nat. Genet. *49*, 1385–1391.
52. Scott, R.A., Scott, L.J., Mägi, R., Marullo, L., Gaulton, K.J., Kaakinen, M., Pervjakova, N., Pers, T.H., Johnson, A.D., Eicher, J.D., et al. (2017). An Expanded Genome-Wide Association Study of Type 2 Diabetes in Europeans. Diabetes *66*, 2888–2902.
53. Purcell, S., Neale, B., Todd-Brown, K., Thomas, L., Ferreira, M.A.R., Bender, D., Maller, J., Sklar, P., de Bakker, P.I.W., Daly, M.J., and Sham, P.C. (2007). PLINK: a tool set for whole-genome association and population-based linkage analyses. Am. J. Hum. Genet. *81*, 559–575.
54. Privé, F., Aschard, H., Carmi, S., Folkersen, L., Hoggart, C., O'Reilly, P.F., and Vilhjálmsson, B.J. (2022). Portability of 245 polygenic scores when derived from the UK Biobank and applied to 9 ancestry groups from the same cohort. Am. J. Hum. Genet. *109*, 373.
55. Florian, P., Vilhja'lmsson, B.J., Aschard, H., and Blum, M.G.B. (2019). Making the Most of Clumping and Thresholding for Polygenic Scores. Am. J. Hum. Genet. *105*, 1213–1221.
56. Chung, W., Chen, J., Turman, C., Lindstrom, S., Zhu, Z., Loh, P.R., Kraft, P., and Liang, L. (2019). Efficient cross-trait penalized regression increases prediction accuracy in large cohorts using secondary phenotypes. Nat. Commun. *10*, 569.
57. Saevarsdottir, S., Olafsdottir, T.A., Ivarsdottir, E.V., Halldorsson, G.H., Gunnarsdottir, K., Sigurdsson, A., Johannesson, A., Sigurdsson, J.K., Juliusdottir, T., Lund, S.H., et al. (2020). FLT3 stop mutation increases FLT3 ligand level and risk of autoimmune thyroid disease. Nature *584*, 619–623.
58. Ng, S.C., Shi, H.Y., Hamidi, N., Underwood, F.E., Tang, W., Benchimol, E.I., Panaccione, R., Ghosh, S., Wu, J.C.Y., Chan, F.K.L., et al. (2017). Worldwide incidence and prevalence of inflammatory bowel disease in the 21st century: a systematic review of population-based studies. Lancet *390*, 2769–2778.
59. Ji, S.-G., Juran, B.D., Mucha, S., Folseraas, T., Jostins, L., Melum, E., Kumasaka, N., Atkinson, E.J., Schlicht, E.M., Liu, J.Z., et al. (2017). Genome-wide association study of primary sclerosing cholangitis identifies new risk loci and quantifies the genetic relationship with inflammatory bowel disease. Nat. Genet. *49*, 269–273.
60. Jostins, L., Ripke, S., Weersma, R.K., Duerr, R.H., McGovern, D.P., Hui, K.Y., Lee, J.C., Schumm, L.P., Sharma, Y., Anderson, C.A., et al. (2012). Host-microbe interactions have shaped the genetic architecture of inflammatory bowel disease. Nature *491*, 119–124.
61. Luo, Y., de Lange, K.M., Jostins, L., Moutsianas, L., Randall, J., Kennedy, N.A., Lamb, C.A., McCarthy, S., Ahmad, T., Edwards, C., et al. (2017). Exploring the genetic architecture of inflammatory bowel disease by whole-genome sequencing identifies association at ADCY7. Nat. Genet. *49*, 186–192.
62. Weissbrod, O., Kanai, M., Shi, H., Gazal, S., Peyrot, W.J., Khera, A.V., Okada, Y., Biobank Japan Project, Martin, A.R., Finucane, H.K., and Price, A.L. (2022). Leveraging fine-mapping and multipopulation training data to improve cross-population polygenic risk scores. Nat. Genet. *54*, 450–458.

The American Journal of Human Genetics, Volume *111*

Supplemental information

shaPRS: Leveraging shared genetic effects across traits or ancestries improves accuracy of polygenic scores

Martin Kelemen, Elena Vigorito, Laura Fachal, Carl A. Anderson, and Chris Wallace

Supplemental notes

shaPRS walkthrough examples

The following three examples may help to explain the application of shaPRS in three illustrative scenarios of SNPs: fully shared, non-shared and partially shared effect between studies.

Fully shared SNP. A SNP whose effect is 100% shared between proximal and adjunct studies. These are frequently null SNPs that have a true effect size of 0 ($\beta_1 = \beta_2 = \beta_{12} = 0$). Here, the estimated lFDR would be close to 1 (π=1), so the shaPRS equation

$$\beta_{shaPRS} = (1 - \pi)\beta_1 + \pi\beta_{12},$$

would simplify to

$$\beta_{shaPRS} \simeq 1 * \beta_{12}.$$

Thus here the final SNP estimate would become close to identical to the meta-analysis (β_{12}).

SNP effect is specific to proximal study. A SNP that only has an effect in the proximal study, but not in the adjunct study. An example of this would be some SNPs in the *NOD2* region in our IBD analyses, which are associated with CD susceptibility but not UC susceptibility. Here, the estimated lFDR would be 0 (π=0). Therefore, the shaPRS equation would simplify to

$$\beta_{shaPRS} \simeq 1 * \beta_1.$$

Thus, the final SNP estimate would become close to the proximal study (β_1).

SNP effect is partially shared between studies. A SNP that has an effect on both phenotypes, but with different magnitudes in the proximal and adjunct studies (which should give rise to a low lFDR for the variant's Cochran's test). Here, the estimated lFDR would be x (π=x), which would be a value between zero and one. Therefore, the shaPRS equation would become

$$\beta_{shaPRS} = (1 - x)\beta_1 + x\beta_{12}.$$

Thus, β_{shaPRS} has an intermediate value between the proximal effect and the meta-analysis effect that depends on the exact degree of effect sharing, specific to that particular SNP.

Missing SNPs and practical application of shaPRS

By default, the shaPRS R package will keep SNPs that are missing in the adjunct data by using their proximal data estimates, which is expected to produce the best overall quality PRS in practical applications. However, for our comparisons we excluded all SNPs that were missing from the adjunct datasets to ensure all methods worked from the same set of SNPs. Incorporating estimates from proximal-only SNPs would have had the effect of adding a constant value to PRS estimates from all methods, which would not have altered the rank order of the methods.

Supplemental figures and legends

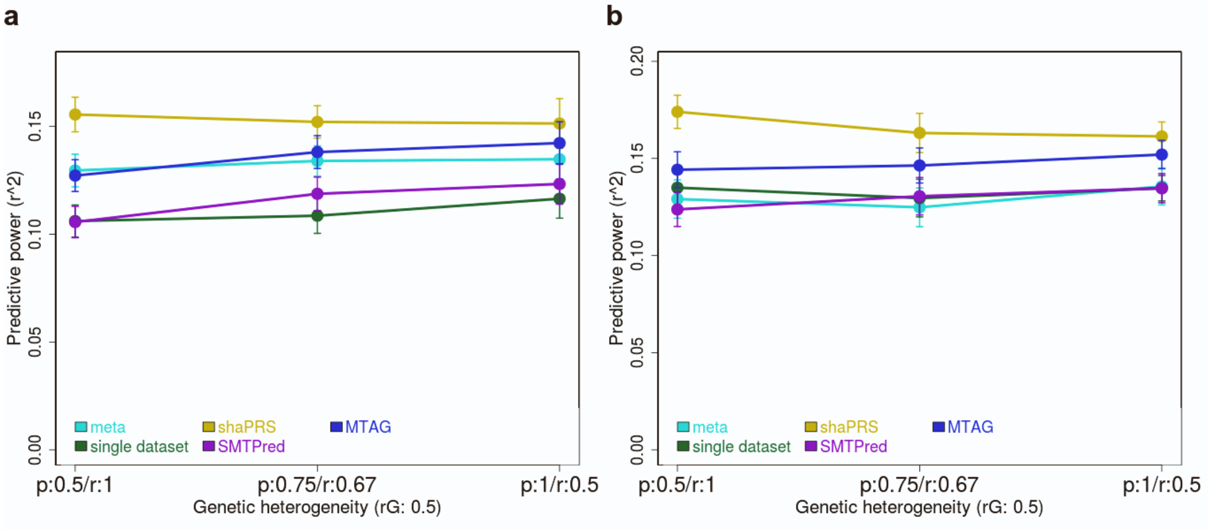

Fig S1: The effect of varying the composition of heterogeneity in the genetic correlation (the proportion of shared causal effects to their correlation) across the five methods. The X-axis shows the three different compositions that were used to generate the same genetic correlation (rG = 0.5). The axis labels are coded as *p/r*, which are the shared fraction of causal SNPs / effect size correlation of these SNPs. The Y-axis represents the squared correlation between the predicted and observed phenotypes on the test set and the error bars represent the standard error of the mean. Meta-analysis (blue) represents the PRS built from combining both phenotypes. 'Single dataset' (green) represents the PRS built from only the individuals from the proximal dataset. shaPRS (yellow) is our method, MTAG (blue) is a method that generates PRS by estimating SNP effect sizes based on constant parameters, and SMTPred (purple) is a method that produces a PRS by balancing the PRS for proximal and adjunct datasets based on their genetic correlation. **a.** Simulation scenario without the extra heterogeneity created by SNPs of large effect. **b.** The same simulation scenario as **a**, with the addition of the extra heterogeneity created by SNPs of large effect which contributed 5% non-shared heritability.

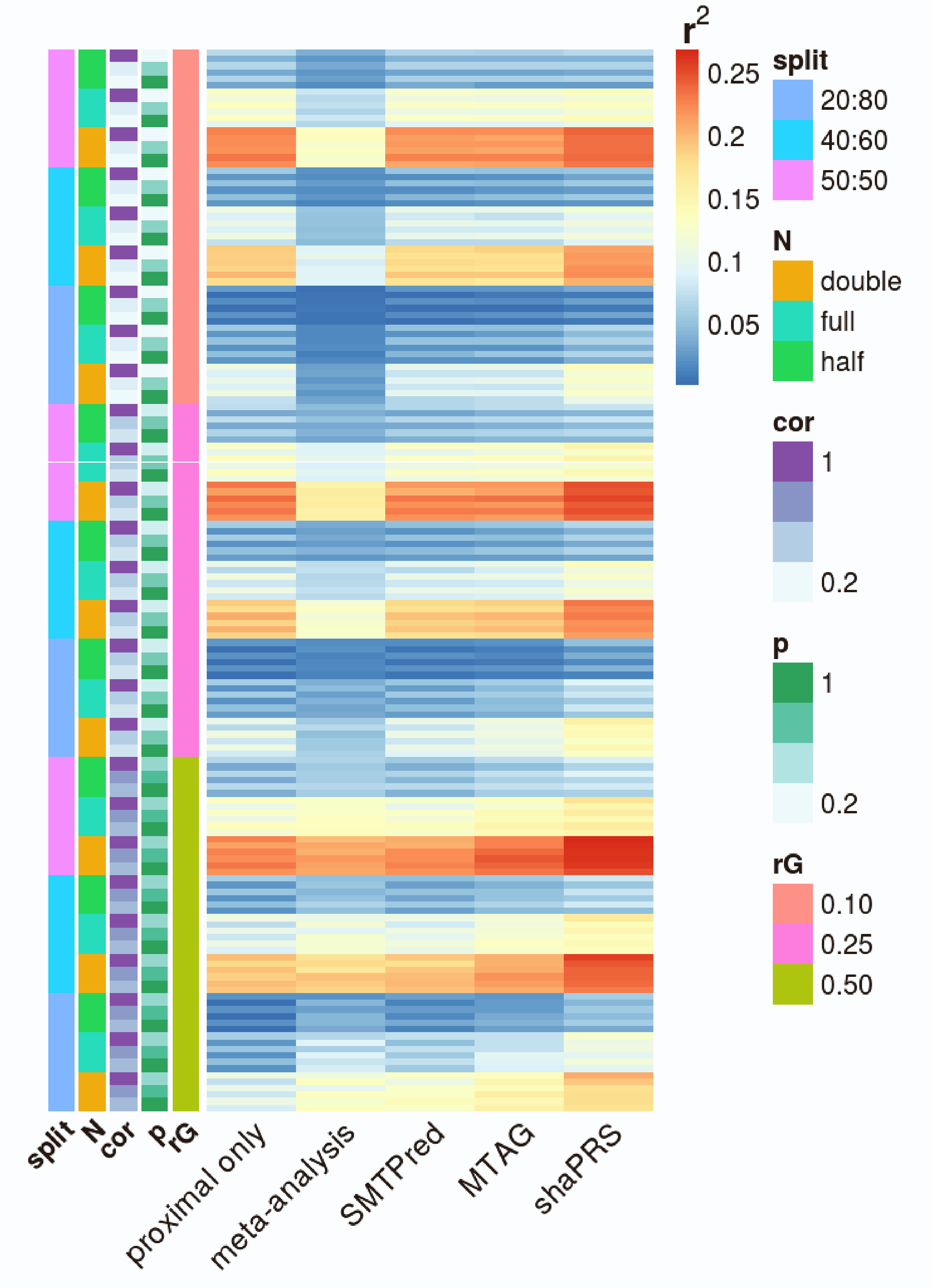

Fig S2: Heatmap of results for all 162 simulation scenarios, colours indicate the squared correlation between simulated and predicted phenotypes for example scenarios, where warmer colours indicate better performance. *N* is the sample size, half, full and double are 7,022, 14,044 and 28,088 individuals, respectively. *p* is the fraction of causal SNPs shared between the proximal and adjunct datasets, *cor* is the correlation of effect sizes between these SNPs. *split* is the ratio of the proximal to adjunct dataset sizes. *rG* is the genetic correlation between the proximal to adjunct datasets.

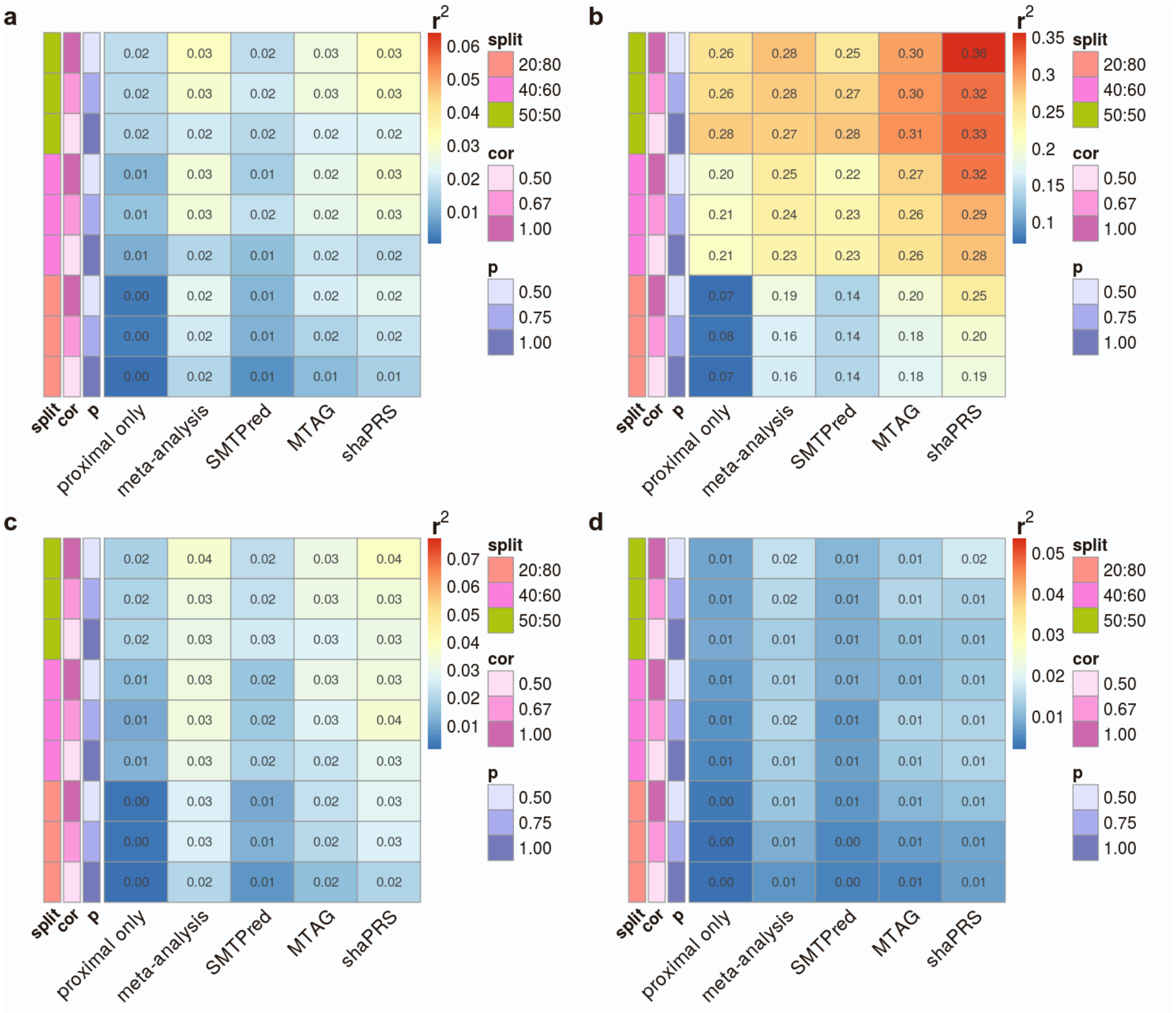

Fig S3: Heatmap of the squared correlation between simulated and predicted phenotypes in the additional 36 scenarios exploring selected parameters. *p* is the fraction of causal SNPs shared between the proximal and adjunct datasets, *cor* is the correlation of effect sizes between these SNPs. *split* is the ratio of the proximal to adjunct dataset sizes. Warmer colours indicate better performance. **a.** Sample size N = 14,044, with a proximal/adjunct sample ratio of 50/50, 40/60 or 20/80, a genetic correlation between proximal and adjunct traits of 0.5 with a heritability of 0.25 from 1,000 causal variants, no extra heterogeneity created by SNPs of large effect. **b.** The same scenario as **a**, with a heritability of 0.75. **b.** **c.** The same scenario as **b**, with a heritability of 0.5 and 3,000 causal SNPs. **d.** The same scenario as **c**, with 5,000 causal SNPs.

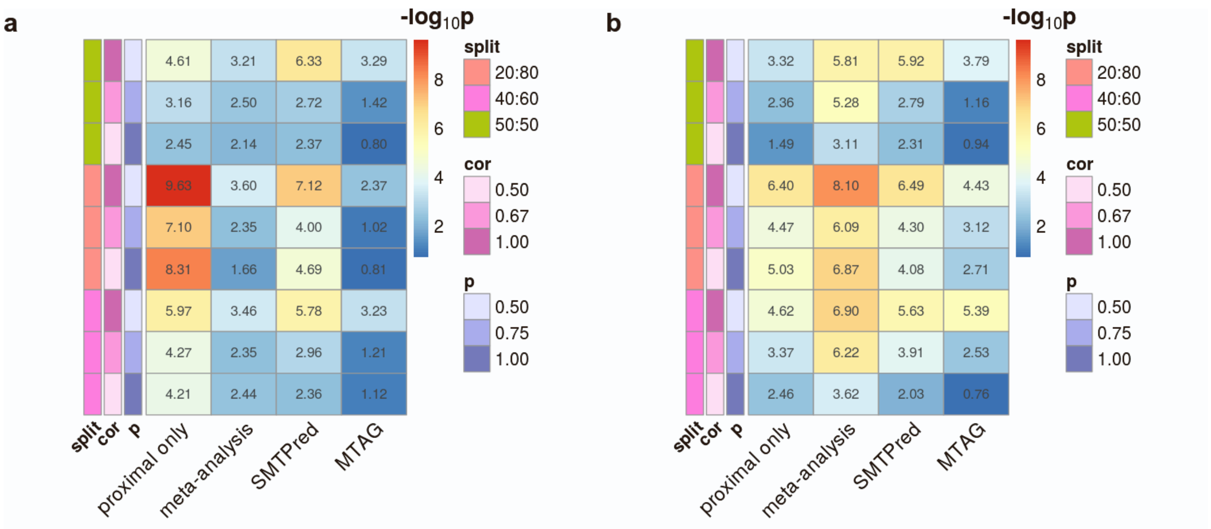

Fig S4: Heatmap of the median ‘r2diff’ -log10(p) model difference between shaPRS and other methods between simulated and predicted phenotypes for selected cross-trait genetic relationships. Warmer colours indicate stronger evidence for a difference between methods. **a.** A genome-wide genetic correlation between proximal and adjunct traits of 0.5 with a heritability of 0.5 from 1,000 causal variants and no extra heterogeneity created by SNPs of large effect. Sample size N = 14,044, with a proximal/adjunct sample ratio of 50/50, 40/60 or 20/80, and where *cor* is the correlation of effect sizes between SNPs and *P (or* $causal_S$*)* is the fraction of causal SNPs shared between the proximal and adjunct datasets. *split* is the ratio of the proximal to adjunct dataset sizes. **b.** The same scenario as **a**, with the addition of extra heterogeneity created by five SNPs of large effect that contributed 5% non-shared heritability. Results across the complete set of simulated scenarios are shown in Fig S2.

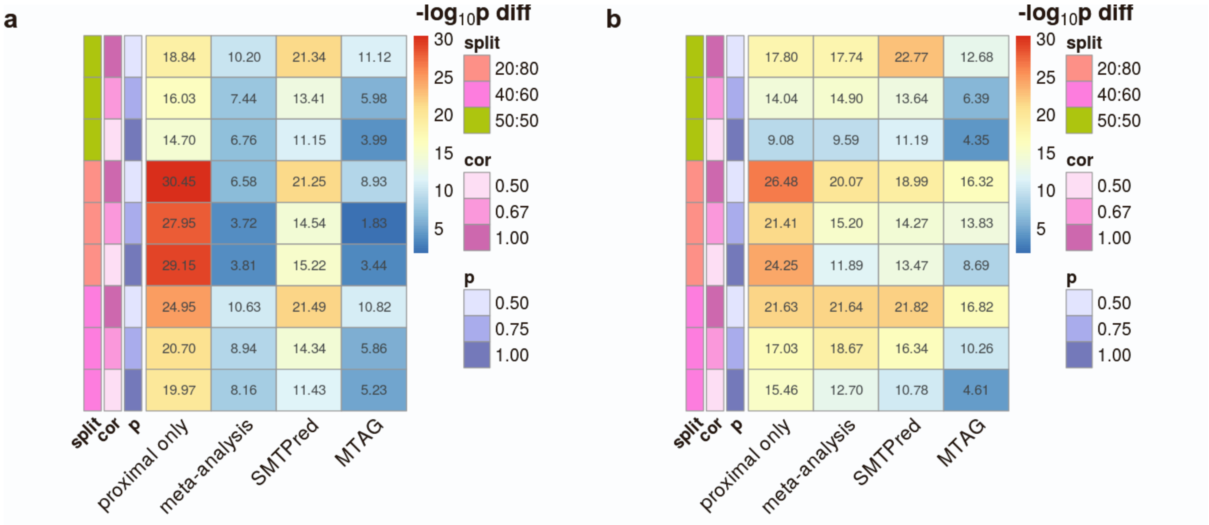

Fig S5: Heatmap of the difference between median -log10(p) values of the likelihood ratio tests of the complex model (which included both shaPRS and the other method) versus the nested model that included either just shaPRS or just the other method for selected cross-trait genetic relationships. Positive values and warmer colours indicate stronger evidence for improving performance by adding shaPRS into the model versus adding the other method. **a.** A genome-wide genetic correlation between proximal and adjunct traits of 0.5 with a heritability of 0.5 from 1,000 causal variants and no extra heterogeneity created by SNPs of large effect. Sample size N = 14,044, with a proximal/adjunct sample ratio of 50/50, 40/60 or 20/80, and where *cor* is the correlation of effect sizes between SNPs and *P (or* $causal_S$*)* is the fraction of causal SNPs shared between the proximal and adjunct datasets. *split* is the ratio of the proximal to adjunct dataset sizes. **b.** The same scenario as **a**, with the addition of extra heterogeneity created by five SNPs of large effect that contributed 5% non-shared heritability. Results across the complete set of simulated scenarios are shown in Fig S2.

Supplemental tables

Table S1 | Range of parameters evaluated in the simulation experiments.

parameter	range		
sample size	7,022, 14,044 and 28,088 training individuals		
phenotype split (proximal/adjunct)	50/50, 40/60 and 20/80		
five large effect SNPs	enabled or disabled		
	**rG**	**shared fraction of causal SNPs**	**effect size correlation**
rG composition	0.1	0.1	1
		0.55	0.182
		1	0.1
	0.25	0.25	1
		0.625	0.4
		1	0.25
	0.5	0.5	1
		0.75	0.667
		1	0.5

Sample size represents the number of individuals used for training the PRS, which were chosen to be approximately half, equal to or double the size of our UC GWAS datasets (N = 4,647 cases and 10,308 controls). Phenotype split represents the percentage of the samples with quantitative phenotypes simulated for each of the two traits, given as proximal/adjunct. The *'five large effect SNPs'* represents the choice to include five highly penetrant SNPs that explained 5% of the non-shared heritability of each trait. *rG* composition represents the different ways genetic correlations were constructed as a product of three different 'shared fraction of causal SNPs' and 'effect size correlation' estimates.

Table S2 | Leveraging information from GWAS studies with different ancestries

Target ancestry	trait	data	information pooling	PRS	r^2	AUC
EUR	T2D	proximal (EUR)	*N/A*	LDpred2	0.0075	0.624
				PRS-CS	0.00958	0.639
		proximal + adjunct	PRS-CSx	PRS-CSx	0.0125	0.658
				PRS-CSx-stage1	0.0114	0.650
			shaPRS	LDpred2	**0.0129**	**0.661**
				PRS-CS	0.0127	0.659
EUR	height	proximal	*N/A*	LDpred2	0.0976	*N/A*
				PRS-CS	0.116	
		proximal + adjunct	PRS-CSx	PRS-CSx	**0.123**	
				PRS-CSx-stage1	0.121	
			shaPRS	LDpred2	0.122	
				PRS-CS	0.122	
EUR	BRCA	proximal	*N/A*	LDpred2	0.00828	0.599
				PRS-CS	0.00529	0.584
		proximal + adjunct	PRS-CSx	PRS-CSx	0.00836	0.602
				PRS-CSx-stage1	0.00684	0.593
			shaPRS	LDpred2	**0.00955**	**0.607**
				PRS-CS	0.00724	0.596
EUR	CAD	proximal	*N/A*	LDpred2	0.00666	0.605
				PRS-CS	**0.0195**	**0.676**
		proximal + adjunct	PRS-CSx	PRS-CSx	0.0179	0.670
				PRS-CSx-stage1	0.0179	0.670
			shaPRS	LDpred2	0.0164	0.663
				PRS-CS	0.0169	0.665
		proximal	*N/A*	LDpred2	0.0115	0.595
				PRS-CS	0.0099	0.588
		proximal	PRS-CSx	PRS-CSx	0.0134	0.603
				PRS-CSx-stage1	0.0113	0.595

EUR	asthma	+ adjunct	shaPRS	LDpred2	**0.0136**	**0.604**
				PRS-CS	0.0123	0.599
AFR	BMI	proximal	*N/A*	LDpred2	0.0025	*N/A*
				PRS-CS	0.0037	
		proximal + adjunct	PRS-CSx	PRS-CSx	0.0210	
				PRS-CSx-stage1	0.0053	
			shaPRS	LDpred2	0.0196	
				PRS-CS	**0.0249**	
AFR	height	proximal	*N/A*	LDpred2	0.0035	*N/A*
				PRS-CS	0.0039	
		proximal + adjunct	PRS-CSx	PRS-CSx	**0.0282**	
				PRS-CSx-stage1	0.0091	
			shaPRS	LDpred2	0.0126	
				PRS-CS	0.0039	
AFR	LDL	proximal	*N/A*	LDpred2	0.0559	*N/A*
				PRS-CS	0.0082	
		proximal + adjunct	PRS-CSx	PRS-CSx	**0.0563**	
				PRS-CSx-stage1	0.0296	
			shaPRS	LDpred2	0.0208	
				PRS-CS	0.0187	

Table of the results of the cross-ancestry analysis that compared the accuracy of six different methods to produce a PRS. **Target ancestry** is the genetic ancestry of the target individuals on whom the final PRS was evaluated. **trait** is the phenotype evaluated. **data** is the summary statistic dataset used for training. Proximal is the GWAS conducted in the target ancestry and proximal+adjunct is the target and adjunct GWAS together. The adjunct GWAS was sourced from Japanese individuals in the case of European target PRS, and European individuals in the case of African target PRS. **information pooling** is the method that was used to pool the information from the proximal and adjunct datasets. *N/A* is when no information pooling took place, *PRS-CSx* is the PRS-CSx method and *shaPRS* is the method presented in this paper. **PRS** is the method that was used to generate the final PRS profiles. *LDpred2* is the PRS generated by the LDpred2-auto method that uses no additional genotype data. *PRS-CS* is the PRS generated by the PRS-CS method that uses no additional genotype data. *PRS-CSx* is the PRS generated by the PRS-CSx method that used validation data to weigh between the target and adjunct PRS. *PRS-CSx-stage1* is the PRS generated by the PRS-CSx method that did not use validation data to weigh between the target and adjunct PRS. $\mathbf{r^2}$ is the squared Pearson correlation coefficient between predicted and observed phenotypes. **AUC** is the area under the receiver operating characteristic curve of the predicted and observed phenotypes. All PRS were evaluated on strictly non-overlapping subsets of the UK Biobank.

Table S3 | shaPRS performance (r^2) relative to other methods in IBD subtypes

Other method	CD	UC
proximal	0.103 (-4%)	0.052 (-22%)
meta-analysis	0.095 (-12%)	0.061 (-6%)
SMTPred	0.100 (-7%)	0.059 (-10%)
MTAG	0.096 (-11%)	0.044 (-39%)
shaPRS	0.107 *(N/A)*	0.065 *(N/A)*

Table of the results for the inflammatory bowel disease subtype analysis that shows the performance improvements achieved by shaPRS relative to other methods. The values in each row are r^2, the squared Pearson correlation coefficient between predicted and observed phenotypes, followed by the percentage difference relative to shaPRS. **CD** is Crohn's disease and **UC** is ulcerative colitis.

Table S4A | CD - shaPRS comparison against other methods

	model difference		LRT of nested vs complex model (p-value)	
**method**	**r2redux p**	**Delong p**	**other**	**shaPRS**
**SMTPred**	0.041	0.23	0.055	2.290E-09
**MTAG**	0.053	0.041	4.550E-07	3.69E-18

Table S4B | UC- shaPRS comparison against other methods

	model difference		LRT of nested vs complex model (p-value)	
**method**	**r2redux p**	**Delong p**	**other**	**shaPRS**
**SMTPred**	0.029	0.044	0.171	1.530E-08
**MTAG**	2.440E-06	5.310E-06	0.138	1.200E-24

Results from the formal evaluation of model difference between shaPRS and other methods for the inflammatory bowel disease subtype. The **model difference** column shows the p-values if there was a difference between shaPRS and the other methods via the ‘r2redux r_diff’ and the pROC’ Delong’ tests, respectively. The **LRT of nested vs complex model** column shows the p-values for a likelihood ratio tests that evaluate if adding **shaPRS** or the **other** (non-shaPRS) PRS onto a nested model improves over the complex model of shaPRS+other. **A.** Crohn's disease and **B.** Ulcerative Colitis.

Table S5A | EUR-EAS asthma - shaPRS comparison against other methods

		method	PRS-CSx	PRS-CSx-stage1
**model difference**	**shaPRS-PRSCS**	**r2redux p**	9.420E-07	0.0026
		**delong p**	3.290E-06	0.00257
	**shaPRS+LDpred2**	**r2redux p**	0.047	4.83E-13
		**delong p**	0.0947	5.64E-12
**LRT of nested vs complex model**	**shaPRS-PRSCS**	**other**	2.11E-37	7.420E-09
		**shaPRS**	0.00296	7.12E-32
	**shaPRS+LDpred2**	**other**	2.02E-16	3.260E-07
		**shaPRS**	9.32E-34	3.28E-81

Table S5B | EUR-EAS height - shaPRS comparison against other methods

		method	PRS-CSx	PRS-CSx-stage1
**model difference**	**shaPRS-PRSCS**	**r2redux p**	1.100E-03	0.387
	**shaPRS+LDpred2**	**r2redux p**	0.821	0.127
**LRT of nested vs complex model**	**shaPRS-PRSCS**	**other**	2.66E-77	1.870E-56
		**shaPRS**	7.05E-34	6.50E-69
	**shaPRS+LDpred2**	**other**	<2.225074e-308	2.600E-297
		**shaPRS**	<2.225074e-308	<2.225074e-308

Table S5C | EUR-EAS T2D - shaPRS comparison against other methods

		method	PRS-CSx	PRS-CSx-stage1
model difference	shaPRS-PRSCS	r2redux p	0.533	6.567e-06
	shaPRS+LDpred2	r2redux p	0.219	2.359e-06
LRT of nested vs complex model	shaPRS-PRSCS	other	1.114e-17	1.106e-11
		shaPRS	1.588e-24	6.481e-56
	shaPRS+LDpred2	other	1.305e-28	1.729e-16
		shaPRS	2.942e-43	1.816e-68

Table S5D | EUR-EAS CAD- shaPRS comparison against other methods

		method	PRS-CSx	PRS-CSx-stage1
model difference	shaPRS-PRSCS	r2redux p	0.005	0.005
	shaPRS+LDpred2	r2redux p	2.992e-04	3.067e-4
LRT of nested vs complex model	shaPRS-PRSCS	other	7.982e-36	5.069e-36
		shaPRS	5.136e-13	3.451e-13
	shaPRS+LDpred2	other	7.314e-53	4.798e-53
		shaPRS	1.251e-17	8.696e-18

Table S5E | EUR-EAS BRCA- shaPRS comparison against other methods

		method	PRS-CSx	PRS-CSx-stage1
**model difference**	**shaPRS-PRSCS**	**r2redux p**	1.069e-05	0.217
	**shaPRS+LDpred2**	**r2redux p**	0.001	7.501e-10
**LRT of nested vs complex model**	**shaPRS-PRSCS**	**other**	2.279e-21	7.383e-07
		**shaPRS**	0.619	5.113e-15
	**shaPRS+LDpred2**	**other**	1.477e-05	0.002
		**shaPRS**	4.290e-28	1.003e-53

Table S5F | EUR-AFR BMI - shaPRS comparison against other methods

		method	PRS-CSx	PRS-CSx-stage1
**model difference**	**shaPRS-PRSCS**	**r2redux p**	1.590E-01	0.00164
	**shaPRS+LDpred2**	**r2redux p**	0.915	0.0207
**LRT of nested vs complex model**	**shaPRS-PRSCS**	**other**	7.11E-03	1.290E-03
		**shaPRS**	1.49E-07	2.62E-16
	**shaPRS+LDpred2**	**other**	2.25E-06	8.810E-04
		**shaPRS**	8.98E-07	2.33E-12

Table S5G | EUR-AFR height - shaPRS comparison against other methods				
		**method**	**PRS-CSx**	**PRS-CSx-stage1**
**model difference**	**shaPRS-PRSCS**	**r2redux p**	9.810E-03	0.25
	**shaPRS+LDpred2**	**r2redux p**	0.000104	0.847
**LRT of nested vs complex model**	**shaPRS-PRSCS**	**other**	4.16E-09	2.32E-06
		**shaPRS**	2.69E-01	8.410E-11
	**shaPRS+LDpred2**	**other**	1.47E-12	3.810E-07
		**shaPRS**	9.96E-01	7.73E-08

Table S5H | EUR-AFR LDL - shaPRS comparison against other methods				
		**method**	**PRS-CSx**	**PRS-CSx-stage1**
**model difference**	**shaPRS-PRSCS**	**r2redux p**	1.130E-11	0.00494
	**shaPRS+LDpred2**	**r2redux p**	9.81E-09	0.0475
**LRT of nested vs complex model**	**shaPRS-PRSCS**	**other**	1.83E-30	4.04E-15
		**shaPRS**	7.94E-01	1.170E-03
	**shaPRS+LDpred2**	**other**	1.01E-27	1.620E-13
		**shaPRS**	2.97E-01	4.11E-05

Table of the results for the formal evaluation of model difference between shaPRS and other methods for the cross-ancestry analyses. The **model difference** row shows the p values if there was a difference between shaPRS and the other methods via the ‘r2redux’ r_diff’ and for binary traits, the pROC’ Delong’ tests, respectively. The **LRT of nested vs complex model** row shows the p-values for a likelihood ratio tests that evaluate if adding **shaPRS** or the **other** PRS onto a nested model improves over the complex model of shaPRS+other. For these cross-ancestry analyses shaPRS was evaluated via both PRS-CS (**shaPRS-PRSCS**) and via LDpred2 (**shaPRS+LDpred2**). **A.** EUR-EAS asthma, **B.** EUR-EAS height, **C.** EUR-EAS T2D, **D.** EUR-EAS CAD, **E.** EUR-EAS BRCA, **F.** EUR-AFR BMI, **G.** EUR-AFR height and **H.** EUR-AFR LDL.

Supplementary Methods: mathematical derivations

1 Test for homogeneity of effects between two studies

Assume we have summary statistics for the same SNP from two studies, $\hat{\beta}_1, \hat{\beta}_2$ estimating the log odds ratios, and σ_1^2, σ_2^2 estimating their variances. We wish to test

$$H_0 : \beta_1 = \beta_2$$

where β_1, β_2 are the estimands of $\hat{\beta}_1, \hat{\beta}_2$.

Now

$$\text{Var}(\hat{\beta}_1 - \hat{\beta}_2) = \sigma_1^2 + \sigma_2^2 - 2\rho\sigma_1\sigma_2$$

where

$$\rho = cor(\hat{\beta}_1, \hat{\beta}_2) = \frac{m_{12}\sqrt{n_1 n_2}}{\sqrt{m_1 m_2 (n_1 + m_1)(n_2 + m_2)}} \tag{1}$$

may be estimated using approximations derived by Lin and Sullivan (2009), with n_1, n_2 the number of cases in studies 1 and 2, m_1, m_2 the number of controls, and m_{12} the number of controls shared between both studies.

Thus a test statistic for homogeneity of effects at the same SNP in two different studies is

$$X^2 = \frac{(\hat{\beta}_1 - \hat{\beta}_2)^2}{\sigma_1^2 + \sigma_2^2 - 2\rho\sigma_1\sigma_2} \sim \chi_1^2$$

This is equivalent to Cochran's Q statistic for two studies, without assuming independence between the studies.

2 shaPRS summary effect estimate

shaPRS requires calculating the combined effect estimate if the two studies had been jointly analysed. We use the standard fixed effects meta analysis to give

$$\hat{\beta}_{12} = \frac{\hat{\beta}_1\sigma_1^{-2} + \hat{\beta}_2\sigma_2^{-2}}{\sigma_1^{-2} + \sigma_2^{-2}} = \frac{\hat{\beta}_1\tau_1 + \hat{\beta}_2\tau_2}{\tau_1 + \tau_2}$$

where to simplify the notation we use the conventional notation for the precision $\tau_. = 1/\sigma_.^2$ with

$$\begin{aligned}\sigma_{12}^2 = \text{Var}(\hat{\beta}_{12}) &= \frac{\tau_1^2\sigma_1^2 + \tau_2^2\sigma_2^2 + \tau_1\tau_2 \,\text{cov}(\hat{\beta}_1, \hat{\beta}_2)}{(\tau_1 + \tau_2)^2} \\ &= \frac{\tau_1 + \tau_2 + \rho\sqrt{\tau_1\tau_2}}{(\tau_1 + \tau_2)^2}\end{aligned}$$

From this and the single study effects, a summary effect is estimated as

$$\bar{\beta} = w\hat{\beta}_1 + (1-w)\hat{\beta}_{12}$$

where $0 < w < 1$ is the lFDR. We have

$$\begin{aligned}
\operatorname{cov}(\hat{\beta}_1, \hat{\beta}_{12}) &= \operatorname{cov}\left(\hat{\beta}_1, \frac{\tau_1\hat{\beta}_1 + \tau_2\hat{\beta}_2}{\tau_1+\tau_2}\right) \\
&= \left(\frac{\tau_1}{\tau_1+\tau_2}\right)\operatorname{cov}(\hat{\beta}_1, \hat{\beta}_1) + \left(\frac{\tau_2}{\tau_1+\tau_2}\right)\operatorname{cov}(\hat{\beta}_1, \hat{\beta}_2) \\
&= \frac{1}{\tau_1+\tau_2} + \frac{\sqrt{\tau_2/\tau_1}}{\tau_1+\tau_2}\rho
\end{aligned}$$

so

$$\begin{aligned}
\bar{\sigma}^2 = \operatorname{Var}\bar{\beta} &= w^2\operatorname{Var}\hat{\beta}_1 + (1-w)^2\operatorname{Var}\hat{\beta}_{12} + 2w(1-w)\operatorname{cov}(\hat{\beta}_1, \hat{\beta}_{12}) \\
&= w^2/\tau_1 + (1-w)^2\frac{\tau_1+\tau_2+\rho\sqrt{\tau_1\tau_2}}{(\tau_1+\tau_2)^2} + 2w(1-w)\frac{1+\rho\sqrt{\tau_2/\tau_1}}{\tau_1+\tau_2} \\
&= \frac{w^2}{\tau_1} + \frac{1-w^2}{\tau_1+\tau_2} + \frac{(1-w)\sqrt{\tau_2}}{(\tau_1+\tau_2)^2\sqrt{\tau_1}}((\tau_1+2\tau_2)w+\tau_1)\rho
\end{aligned}$$

3 Correlation between shaPRS summary effect at two SNPs

In order to calculate a PRS we also need an estimate of the correlation between shaPRS summary effects $\bar{\beta}_A, \bar{\beta}_B$ at two SNPs in LD, A and B, for LD-aware polygenic score methods. From here on we will use subscripts A, B to denote quantities relating to SNPs A and B, and $1, 2$ to denote quantities relating to studies 1 and 2, possibly in combination.

$$\begin{aligned}
\operatorname{cov}(\bar{\beta}_A, \bar{\beta}_B) &= cov\left[\left(w_A\hat{\beta}_{A1} + (1-w_A)\hat{\beta}_{A12}\right), \left(w_B\hat{\beta}_{B2} + (1-w_B)\hat{\beta}_{B12}\right)\right] \\
&= cov\left[\left(w_A\hat{\beta}_{A1} + (1-w_A)\frac{\tau_{A1}\hat{\beta}_{A1}+\tau_{A2}\hat{\beta}_{A2}}{\tau_{A1}+\tau_{A2}}\right), \left(w_B\hat{\beta}_{B2} + (1-w_B)\frac{\tau_{B1}\hat{\beta}_{B1}+\tau_{B2}\hat{\beta}_{B2}}{\tau_{B1}+\tau_{B2}}\right)\right] \\
&= \left(\frac{\tau_{A1}+w_A\tau_{A2}}{\tau_{A1}+\tau_{A2}}\right)\left(\frac{\tau_{B1}+w_B\tau_{B2}}{\tau_{B1}+\tau_{B2}}\right)\operatorname{cov}(\hat{\beta}_{A1}, \hat{\beta}_{B1}) + \\
&\quad \left(\frac{\tau_{A1}+w_A\tau_{A2}}{\tau_{A1}+\tau_{A2}}\right)\left(\frac{(1-w_B)\tau_{B2}}{\tau_{B1}+\tau_{B2}}\right)\operatorname{cov}(\hat{\beta}_{A1}, \hat{\beta}_{B2}) + \\
&\quad \left(\frac{(1-w_A)\tau_{A2}}{\tau_{A1}+\tau_{A2}}\right)\left(\frac{\tau_{B1}+w_B\tau_{B2}}{\tau_{B1}+\tau_{B2}}\right)\operatorname{cov}(\hat{\beta}_{A2}, \hat{\beta}_{B1}) + \\
&\quad \left(\frac{(1-w_A)\tau_{A2}}{\tau_{A1}+\tau_{A2}}\right)\left(\frac{(1-w_B)\tau_{B2}}{\tau_{B1}+\tau_{B2}}\right)\operatorname{cov}(\hat{\beta}_{A2}, \hat{\beta}_{B2}) \qquad (2)
\end{aligned}$$

Standard results give covariances for different SNPs in the same study (Burren *et al.*, 2014)

$$\mathrm{cov}(\hat{\beta}_{A1}, \hat{\beta}_{B1}) = r_1/\sqrt{\tau_{A1}\tau_{B1}}$$
$$\mathrm{cov}(\hat{\beta}_{A2}, \hat{\beta}_{B2}) = r_2/\sqrt{\tau_{A2}\tau_{B2}}$$

where r_i denotes the correlation between genotypes at A and B in study i, or the same SNPs in different studies (Lin and Sullivan, 2009)

$$\mathrm{cov}(\hat{\beta}_{A1}, \hat{\beta}_{A2}) = \rho/\sqrt{\tau_{A1}\tau_{B1}}$$
$$\mathrm{cov}(\hat{\beta}_{B1}, \hat{\beta}_{B2}) = \rho/\sqrt{\tau_{A2}\tau_{B2}}$$

3.1 Non-overlapping samples between studies

If the different studies have no overlap, $\rho = 0$ and the covariance between estimates from different studies (same or different SNPs) is 0. From (2) then, in the case of no overlapping samples, but possibly different populations (so $r_1 \neq r_2$)

$$\mathrm{cov}(\bar{\beta}_{A1}, \bar{\beta}_{B2}) = \left(\frac{\tau_{A1} + w_A\tau_{A2}}{\tau_{A1} + \tau_{A2}}\right)\left(\frac{\tau_{B1} + w_B\tau_{B2}}{\tau_{B1} + \tau_{B2}}\right)\frac{r_1}{\sqrt{\tau_{A1}\tau_{B1}}} + \left(\frac{(1 - w_A)\tau_{A2}}{\tau_{A1} + \tau_{A2}}\right)\left(\frac{(1 - w_B)\tau_{B2}}{\tau_{B1} + \tau_{B2}}\right)\frac{r_2}{\sqrt{\tau_{A2}\tau_{B2}}}$$

and

$$\begin{aligned}\mathrm{cor}(\bar{\beta}_{A1}\bar{\beta}_{B2}) &= \frac{\mathrm{cov}(\bar{\beta}_{A1}, \bar{\beta}_{B2})}{\sqrt{\bar{\sigma}_A\bar{\sigma}_B}} \\ &= \frac{\sqrt{\tau_{A1}\tau_{A2}\tau_{B1}\tau_{B2}}(1 - w_A)(1 - w_B)r_2 + (\tau_{A1} + w_A\tau_{A2})(\tau_{B1} + w_B\tau_{B2})r_1}{\sqrt{(\tau_{A1} + \tau_{A2})(\tau_{B1} + \tau_{B2})(\tau_{A1} + w_A^2\tau_{A2})(\tau_{B1} + w_B^2\tau_{B2})}}\end{aligned}$$

When the two studies reflect the same populations, so $r_1 = r_2 = r$,

$$\mathrm{cor}(\bar{\beta}_{A1}\bar{\beta}_{B2}) = r \times \frac{\sqrt{\tau_{A1}\tau_{A2}\tau_{B1}\tau_{B2}}(1 - w_A)(1 - w_B) + (\tau_{A1} + w_A\tau_{A2})(\tau_{B1} + w_B\tau_{B2})}{\sqrt{(\tau_{A1} + \tau_{A2})(\tau_{B1} + \tau_{B2})(\tau_{A1} + w_A^2\tau_{A2})(\tau_{B1} + w_B^2\tau_{B2})}}$$

Note that this may be < 1.

3.2 Overlapping samples between studies

Where studies do have overlapping samples, we assume they are from the same population so $r_1 = r_2 = r$, and we follow the Appendix in Lin and Sullivan (2009) to estimate covariances between different SNPs in different studies. Switching for now to the notation there `https://www.ncbi.nlm.nih.gov/pmc/articles/PMC2790578/#app1`, much of the same results hold, and we have

$$\mathrm{cov}(\hat{\theta}_k, \theta_l) \simeq I_k^{-1}\,\mathrm{cov}\{U_k(\theta_k)U_l(\theta_l)\}I_l^{-1}(\theta_l) \tag{3}$$

where $\hat{\theta}_k$ is the root of the score function

$$U_k(\theta_k) = \sum_{i=1}^{N_k} \left(Y_i - \frac{e^{\alpha_k + \beta_k' X_i}}{1 + e^{\alpha_k + \beta_k' X_i}} \right) \tilde{X}_i$$

where N_k is the total sample size, $\tilde{X}_i$ is the covariate matrix augmented with a column of 1s and $\mathrm{Var}(\theta_k) \simeq I_k^{-1}(\theta_k)$ with

$$I_k(\theta_k) = \sum_{i=1}^{N_k} \frac{e^{\alpha_k + \beta_k' X_i}}{(1 + e^{\alpha_k + \beta_k' X_i})^2} \tilde{X}_i \tilde{X}_i{}'$$

Unlike Lin and Sullivan (2009), j, k now refer to **different** SNPs in different studies (eg $j = A1, k = B2$), so $I_k \neq I_l$. Under the null hypothesis, we assume $\beta_k = \beta_l = 0$, allowing some simplification.

$$\begin{aligned}
I_k(\theta_k) &\simeq \frac{N_k e^{\alpha_k}}{(1 + e^{\alpha_k})^2} N_k^{-1} \begin{pmatrix} n_k & \sum X_{ki} \\ \sum X_{ki} & \sum X_{ki}^2 \end{pmatrix} \\
&\simeq \frac{N_k e^{\alpha_k}}{(1 + e^{\alpha_k})^2} \begin{pmatrix} 1 & 2f_k \\ 2f_k & 2f_k(1 + f_k) \end{pmatrix} \\
I_k^{-1}(\theta_k) &\simeq \frac{(1 + e^{\alpha_k})^2}{N_k e^{\alpha_k}} \left(\frac{1}{2f_k(1 + f_k)} - 4f_k^2 \right) \begin{pmatrix} 2f_k(1 + f_k) & -2f_k \\ -2f_k & 1 \end{pmatrix}
\end{aligned}$$

where n_k is cases in study k, f_k is the minor allele frequency for the SNP tested in study k, and similarly for I_l. The approximation to $\mathrm{cov}\{U_k(\theta_k), U_l(\theta_l)\}$ also differs

$$\begin{aligned}
\mathrm{cov}\{U_k(\theta_k), U_l(\theta_l)\} &\simeq \sum_i^{N_{lk}} \left(Y_i - \frac{e^{\alpha_k}}{1 + e^{\alpha_k}} \right) \left(Y_i - \frac{e^{\alpha_l}}{1 + e^{\alpha_l}} \right) \\
&\simeq \frac{e^{\alpha_k + \alpha_l}}{(1 + e^{\alpha_k})(1 + e^{\alpha_l})} \begin{pmatrix} N_{lk} & \sum X_{li} \\ \sum X_{ki} & \sum X_{li} X_{ki} \end{pmatrix} \\
&\simeq \frac{N_{lk} e^{\alpha_k + \alpha_l}}{(1 + e^{\alpha_k})(1 + e^{\alpha_l})} \begin{pmatrix} 1 & 2f_l \\ 2f_k & 2r\sqrt{f_k f_l (1 - f_k)(1 - f_l)} + 4 f_k f_l \end{pmatrix}
\end{aligned}$$

where N_{lk} is the number of shared subjects between studies l, k. Thus from (3)

$$\mathrm{cov}(\hat{\theta}_k, \hat{\theta}_l) \simeq \frac{(1 + e^{\alpha_k})(1 + e^{\alpha_l}) N_{kl}}{4 N_k N_l f_k f_l (1 - f_k)(1 - f_l)} \begin{pmatrix} 4 f_k f_l (F + f_k f_l - f_k - f_l + 1) & -2F f_k \\ -2F f_l & F \end{pmatrix}$$

where $F = 2r\sqrt{f_k f_l (1 - f_k)(1 - f_l)}$ so

$$\begin{aligned}
\mathrm{cov}(\hat{\beta}_k, \hat{\beta}_l) &\simeq \frac{(1 + e^{\alpha_k})(1 + e^{\alpha_l}) N_{kl}}{4 N_k N_l f_k f_l (1 - f_k)(1 - f_l)} 2r\sqrt{f_k f_l (1 - f_k)(1 - f_l)} \\
&= \frac{(1 + e^{\alpha_k})(1 + e^{\alpha_l}) N_{kl}}{2 N_k N_l \sqrt{f_k f_l (1 - f_k)(1 - f_l)}} r
\end{aligned}$$

The MAF enter here because variance of the genotype is a function of MAF. We know (Giambartolomei *et al.*, 2014) that

$$\operatorname{Var}\hat{\beta}_k \simeq 1/\tau_k = \frac{(n_k + m_k)}{n_k m_k \times 2 f_k(1-f_k)}$$

so we can substitute

$$f_k(1-f_k) = \frac{(n_k+m_k)\tau_k}{2n_k m_k}$$

and we also have $e^{\alpha_k} \simeq n_k/m_k$ (Lin and Sullivan, 2009) where m_k is the number of controls in study k, so that (returning to the notation used in this manuscript)

$$\begin{aligned}
\operatorname{cov}(\hat{\beta}_{A1}, \hat{\beta}_{B2}) &\simeq \frac{r m_{12}\sqrt{n_1 n_2}}{\sqrt{m_1 m_2 (n_1+m_1)(n_2+m_2)\tau_{A1}\tau_{B2}}} \\
\operatorname{cor}(\hat{\beta}_{A1}, \hat{\beta}_{B2}) &\simeq \frac{\operatorname{cov}(\hat{\beta}_{A1}, \hat{\beta}_{B2})}{\sqrt{\sigma^2_{A1}\sigma^2_{B1}}} \\
&\simeq \frac{r m_{12}\sqrt{n_1 n_2}}{\sqrt{m_1 m_2 (n_1+m_1)(n_2+m_2)}} \\
&\simeq r\rho
\end{aligned}$$

and similarly

$$\operatorname{cov}(\hat{\beta}_{A2}, \hat{\beta}_{B1}) \simeq \frac{r\rho}{\sqrt{\tau_{A2}\tau_{B1}}}$$

With this, from (2) we can easily estimate

$$\begin{aligned}
\operatorname{cov}(\bar{\beta}_A, \bar{\beta}_B) = & \left(\frac{\tau_{A1} + w_A\tau_{A2}}{\tau_{A1}+\tau_{A2}}\right)\left(\frac{\tau_{B1}+w_B\tau_{B2}}{\tau_{B1}+\tau_{B2}}\right)\frac{r}{\sqrt{\tau_{A1}\tau_{B1}}} + \\
& \left(\frac{\tau_{A1} + w_A\tau_{A2}}{\tau_{A1}+\tau_{A2}}\right)\left(\frac{(1-w_B)\tau_{B2}}{\tau_{B1}+\tau_{B2}}\right)\frac{r\rho}{\sqrt{\tau_{A1}\tau_{B2}}} + \\
& \left(\frac{(1-w_A)\tau_{A2}}{\tau_{A1}+\tau_{A2}}\right)\left(\frac{\tau_{B1}+w_B\tau_{B2}}{\tau_{B1}+\tau_{B2}}\right)\frac{r\rho}{\sqrt{\tau_{A2}\tau_{B1}}} + \\
& \left(\frac{(1-w_A)\tau_{A2}}{\tau_{A1}+\tau_{A2}}\right)\left(\frac{(1-w_B)\tau_{B2}}{\tau_{B1}+\tau_{B2}}\right)\frac{r}{\sqrt{\tau_{A2}\tau_{B2}}}
\end{aligned}$$

References

Burren, O. S., Guo, H., and Wallace, C. (2014). VSEAMS: A pipeline for variant set enrichment analysis using summary GWAS data identifies IKZF3, BATF and ESRRA as key transcription factors in type 1 diabetes. *Bioinformatics*, **30**(23), 3342–3348.

Giambartolomei, C., Vukcevic, D., Schadt, E. E., Franke, L., Hingorani, A. D., Wallace, C., and Plagnol, V. (2014). Bayesian Test for Colocalisation between Pairs of Genetic Association Studies Using Summary Statistics. *PLOS Genetics*, **10**(5), e1004383.

Lin, D.-Y. and Sullivan, P. F. (2009). Meta-analysis of genome-wide association studies with overlapping subjects. *Am. J. Hum. Genet.*, **85**(6), 862–872.
